# Label-Free Investigations on the G Protein Dependent Signaling Pathways of Histamine Receptors

**DOI:** 10.3390/ijms22189739

**Published:** 2021-09-09

**Authors:** Ulla Seibel-Ehlert, Nicole Plank, Asuka Inoue, Guenther Bernhardt, Andrea Strasser

**Affiliations:** 1 Institute of Pharmacy, Faculty of Chemistry and Pharmacy, University of Regensburg, 93040 Regensburg, Germany; nicole.plank@ur.de (N.P.); guenther.bernhardt@ur.de (G.B.); 2 Department of Pharmacological Sciences, Tohoku University, Sendai 980-8578, Japan; iaska@tohoku.ac.jp

**Keywords:** label-free, dynamic mass redistribution (DMR), G protein coupled receptors (GPCRs), histamine receptors, signaling pathways, G protein inhibitors, G protein knock-out

## Abstract

G protein activation represents an early key event in the complex GPCR signal transduction process and is usually studied by label-dependent methods targeting specific molecular events. However, the constrained environment of such “invasive” techniques could interfere with biological processes. Although histamine receptors (HRs) represent (evolving) drug targets, their signal transduction is not fully understood. To address this issue, we established a non-invasive dynamic mass redistribution (DMR) assay for the human H_1–4_Rs expressed in HEK cells, showing excellent signal-to-background ratios above 100 for histamine (HIS) and higher than 24 for inverse agonists with pEC_50_ values consistent with literature. Taking advantage of the integrative nature of the DMR assay, the involvement of endogenous Gα_q/11_, Gα_s_, Gα_12/13_ and Gβγ proteins was explored, pursuing a two-pronged approach, namely that of classical pharmacology (G protein modulators) and that of molecular biology (Gα knock-out HEK cells). We showed that signal transduction of hH_1–4_Rs occurred mainly, but not exclusively, via their canonical Gα proteins. For example, in addition to Gα_i/o_, the Gα_q/11_ protein was proven to contribute to the DMR response of hH_3,4_Rs. Moreover, the Gα_12/13_ was identified to be involved in the hH_2_R mediated signaling pathway. These results are considered as a basis for future investigations on the (patho)physiological role and the pharmacological potential of H_1–4_Rs.

## 1. Introduction

G protein-coupled receptors (GPCRs), also termed seven-transmembrane-domain receptors (7TMs), are integral membrane proteins that transduce a broad variety of extracellular stimuli, ranging from photons and various small molecules to polypeptides, into the cell. As the largest superfamily of proteins in the human genome, GPCRs are involved in many (patho)physiological processes and represent important drug targets in the treatment of numerous diseases [1,2]. Canonical GPCR signal transduction occurs by binding of an agonist to a receptor, stabilizing an active receptor conformation and allowing the receptor to activate heterotrimeric G proteins, composed of Gα, Gβ and Gγ subunits. Upon GPCR activation, the G proteins dissociate from the receptor and split up into Gα and Gβγ subunits. Subsequently, both can modulate specific downstream effectors. The Gα proteins are divided into four major classes (Gα_q/11_, Gα_s_, Gα_i/o_ and Gα_12/13_), based on sequence similarity and their functional properties [3] and are predominantly associated with certain events in the signaling cascade, such as increase in intracellular Ca^2+^ and IP_3_ (Gα_q/11_), in- or decrease in cAMP level (Gα_s_, Gα_i/o_, respectively) or activation of Rho GTPase (Gα_12/13_) [3,4]. By contrast, the effects of the Gβγ subunit are more diffuse [5,6]. Historically, GPCR signaling was assumed to occur via activation of a single class of Gα proteins, and therefore the receptors were typically classified accordingly [7]. However, scientific progress has revealed a complex network of signaling events including pleiotropic G protein (in-)dependent signaling, constitutive activity, biased agonism, receptor (hetero-)oligomerization and cross talk (reviewed in [8,9,10,11]).

To study these processes, a wide range of microtiter assay techniques are available [12,13,14]. For example, events very proximal to ligand binding, such as ligand induced conformational rearrangement of GPCRs [15], and activation and recruitment of (chimeric) G proteins [16] or β-arrestins [17] can be monitored. More distal in the signaling cascade, protein–protein interactions can be analyzed [18] as well as changes in the levels of second messengers (e.g., IP_3_, Ca^2+^ and cAMP [19,20,21,22]) and the expression of gene reporters [20,23]. Despite numerous advantages, these “invasive” label-dependent methods spotlight only individual events in the complex GPCR signaling cascade and can produce system bias [24]. Moreover, the modification and co-expression of tagged proteins could alter the results. Another issue is that analyses of individual processes in the signaling cascade (yielding different readouts) require different experimental conditions and is often carried out in models of different cellular backgrounds (tissue bias). This makes the comparison between such results difficult.

**Figure 1:**
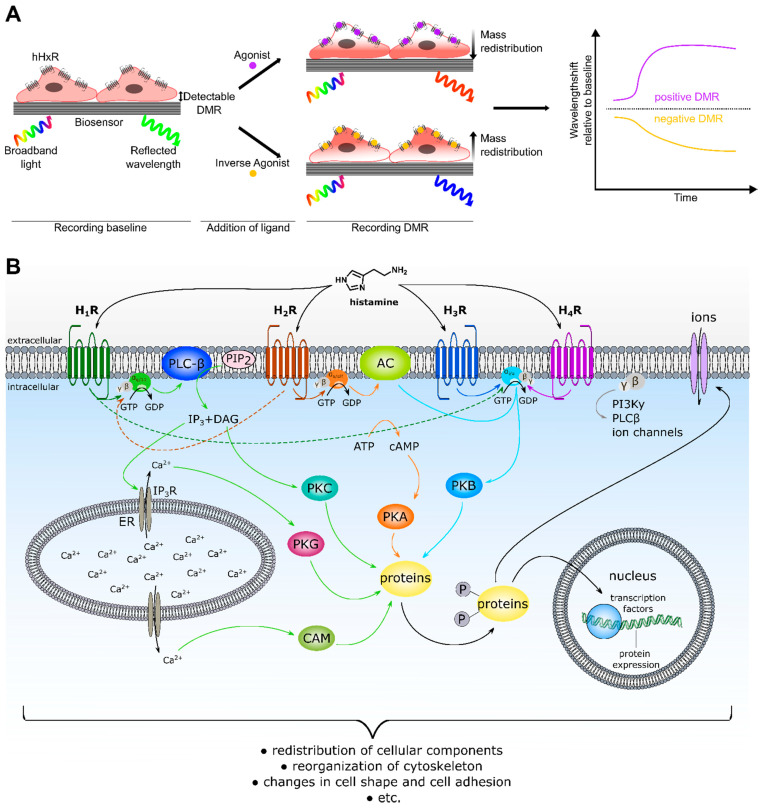
Schematic illustrations of the principle of the DMR assay and canonical signal transduction of histamine H_1–4_Rs. (**A**) The label-free DMR technology detects changes in the refractive index caused by mass redistribution inside a cell, triggered by receptor stimulation, relative to a baseline. Alteration of the refractive index is measured with a biosensor, integrated in each well of a microplate (adapted from Schröder et al. [38]). (**B**) Schematic summary of the signal transduction of H_1–4_Rs according to IUPHAR [56] and Panula et al. [57] (adapted from Panula et al. [57]). Canonical Gα protein signaling is indicated by solid lines. Involvement of secondary Gα proteins is indicated by dashed lines. AC, adenylyl cyclase; CAM, calcium-modulated protein; CTX, cholera toxin; DAG, diacylglycerol; IP_3_, inositol-1,4,5-trisphosphate; PI3Kγ, phosphoinositide 3-kinase-γ; PIP_2_, phosphatidylinositol-4,5-bisphosphate; PKA, protein kinase A; PKB, protein kinase B; PKC, protein kinase C; PLC-β, phospholipase C-β; PTX, pertussis toxin.

Label-free approaches represent powerful “non-invasive” alternatives, able to capture the whole cellular response triggered by GPCR activation, independent of the signaling pathways involved and without the need to constrain specific experimental conditions. Therefore, label-free approaches such as optical dynamic mass redistribution (DMR) are gaining increasing attention both in drug discovery and GPCR signal transduction studies [25,26,27,28]. In a DMR assay, the measurement is based on the change of the refractive index near the biosensor (Figure 1A). This change is caused by a reorganization of cellular components, accompanied by a morphological rearrangement of the cells. In GPCR research, this is triggered by stimulation of a receptor with a ligand [29,30]. As the change in refractive index is measured relative to a baseline, the DMR response can be positively or negatively deflected, depending on both the cell model and the receptor-ligand combination used. Therefore, the DMR signal is a holistic response, reflecting multiple cellular events downstream of receptor activation. Such dynamic response profiles are used to quantify various types of ligand action, including full, partial and inverse agonism, antagonism and allosteric modulation in both native [31,32] and recombinant [33] expression systems. However, due to the complexity of the DMR response, it is difficult to identify which events are reflected by the DMR signal, a phenomenon referred to as a “black box” [34]. Although certain characteristics of the signaling profiles of GPCRs in label-free responses were attributed to the activation of distinct classes of Gα proteins [26,29,35,36], there is also evidence to the contrary [37]. Application of G protein modulators such as pertussis toxin (PTX), FR900359 (FR) and cholera toxin (CTX) [37,38,39] can be used to dissect these G protein dependent signals [37]. PTX selectively and irreversibly inactivates the Gα_i/o_ protein by ADP-ribosylation at the Gα-subunit [20,33,37,40,41,42]. CTX locks the Gα_s_ protein in its GTP bound state by irreversible ADP-ribosylation, leading to a permanent activation of the Gα_s_ protein, which is in turn uncoupled and no longer available for GPCR recruitment [31,32,33,37,43]. As ribosylation leads to a maximum activation of the Gα_s_ protein rather than inhibition of the Gα_s_ protein, the results should be interpreted with caution. FR (alias UBO-QIC) selectively silences Gα_q/11_ signaling by blocking the GDP-release in the Gα subunit [18,41,44,45,46,47], which is a mandatory step in G protein activation. The advantage of such studies is that they can be performed with the same cell model under identical experimental conditions, thereby precluding cell bias. Such investigations have already been performed for a variety of GPCRs [37,38,47,48,49,50,51,52,53]. Another approach to investigate the involvement of G proteins in signal transduction is to prevent their expression by knocking out the corresponding genes [41,54,55]. An advantage of the knock-out strategy over the administration of G protein modulators is the chance that the G protein can be switched off more precisely. In this study, both approaches were followed to investigate the G protein signaling pathways of histamine hH_1–4_ receptors by DMR.

Histamine receptors (HRs) represent important drug targets in the treatment of disorders, such as allergy and reflux diseases [58]. They transmit their signals predominantly via three classes of G proteins: H_1_ via Gα_q/11_, H_2_ via Gα_s_ and H_3_ + H_4_ via Gα_i/o_ (Figure 1B). However, for the H_1_ and H_2_ receptors, evidence is emerging for promiscuous activation of Gα proteins [59,60,61]. By contrast, less information is available on the involvement of non-canonical G protein subunits in the signal transduction processes of the H_3,4_Rs. The first aim of the study was to establish a DMR assay for the entire histamine receptor family to compare the signaling patterns of the H_1–4_Rs in the same experimental setup. For this purpose, the four human receptor subtypes (hH_1–4_Rs) were stably expressed in HEK cells. HEK cells were chosen as they constitutively express the four relevant Gα classes (Gα_s_, Gα_q/11_, Gα_12/13_ and Gα_i/o_) at comparable levels [62]. The contribution of G proteins to the integrated DMR response of hH_1–4_Rs was investigated by pursuing two different approaches. Firstly, in a classical pharmacological approach the G protein signaling pathways in HEK hH_1–4_R cells were silenced using G protein modulators (PTX, CTX, FR and gallein). Secondly, in a molecular biological approach CRISPR/Cas 9 modified Gα knock-out HEK cells (ΔGα_x_ HEK) lacking either the Gα_s/l_ (ΔGα_s/l_ HEK) [63], the Gα_q/11_ (ΔGα_q/11_ HEK) [44] or the Gα_12/13_ (ΔGα_12/13_ HEK) [64] gene were stably transfected with hH_1–4_Rs. Moreover, cells lacking six Gα proteins (ΔGα_s/l, q/11, 12/13_ = ΔGα_six_ HEK) [41], stably expressing the hH_1–4_Rs were used. The results for both approaches were compared and discussed with respect to the impact of G protein inactivation on the hH_1–4_R mediated DMR response.

## 2. Results and Discussion

### 2.1. Characterization of HEK hH_1–4_R Cells

To investigate the effect of endogenously expressed G proteins on the DMR response, HEK hH_1–4_R cells were generated. For this purpose, the human histamine H_1_, H_2_, H_3_ or H_4_ receptor (hH_1–4_Rs) was inserted into a pIRESneo3 vector encoding the signaling peptide (SP) of the murine 5-HT_3A_ receptor and a FLAG tag to give the pIRESneo3-SP-FLAG-hH_1–4_R constructs. Both parental and ΔGα_x_ HEK cells were stably transfected with these constructs to give HEK hH_1–4_R and ΔGα_x_ HEK hH_1–4_R cells. For HEK hH_1–4_R cells, single clones of the stable transfectants were picked, selected, and screened by DMR for the highest signal elicited by 100 µM histamine (data not shown; (structure is presented in supplementary Figure S1). The expression of the hH_1–4_Rs in HEK cells was confirmed by radioligand saturation binding using live cells (Supplementary Figure S2). For the characterization of the ΔGα_x_ HEK hH_1–4_R cells see Section 2.4.1 and supplementary Figure S3. The expression levels of hH_1–4_Rs in HEK hH_1–4_R cells were calculated using B_max_ and the specific activity (a_s_) of the corresponding radioligand and the cell number (Table 1). Despite identical receptor cloning and transfection procedures of the hH_1–4_Rs, the expression level of hH_3_R and hH_4_R was lower compared to the hH_1_R and hH_2_R. The pK_d_ values determined for the respective radioligands at HEK hH_1–4_R cells were in good agreement with literature data (Table 1).

To further characterize the HEK hH_1–4_R cells, radioligand competition binding experiments were performed with histamine (HIS) and one receptor-specific, inverse agonist (diphenhydramine (DPH) at the hH_1_R, famotidine (FAM) at the hH_2_R, pitolisant (PIT) at the hH_3_R and thioperamide (THIO) at the hH_4_R) using live cells (structures are presented in supplementary Figure S1). The displacement curves are shown in supplementary Figure S4 and the pK_i_ values are summarized in Table 2. As expected, HIS had a markedly higher affinity to hH_3,4_Rs compared to hH_1,2_Rs (Table 2). In the literature, pK_i_ values were determined either with cell membranes/homogenates or with live cells. In general, the pK_i_ values determined for HIS and the inverse agonists using HEK hH_2–4_R cells were in the same range as reported in the literature with live cells (Supplementary Table S1). To the best of our knowledge, pK_i_ values for HIS at the hH_1_R in live cells have not yet been reported. Compared to reference values from membranes/homogenates ranging between 4.3–5.9 [65,66,67], the affinity of HIS for the hH_1_R was lower in live cells (pK_i_ = 3.37 ± 0.29). The use of live cells in comparison to membrane preparations has a marked influence of the “apparent affinity” of ligands, especially in the case of agonists [68].

### 2.2. Establishment of a DMR Assay Using HEK hH_1–4_R Cells

#### 2.2.1. Stimulation of HEK hH_1–4_R Cells with Histamine

**Figure 2:**
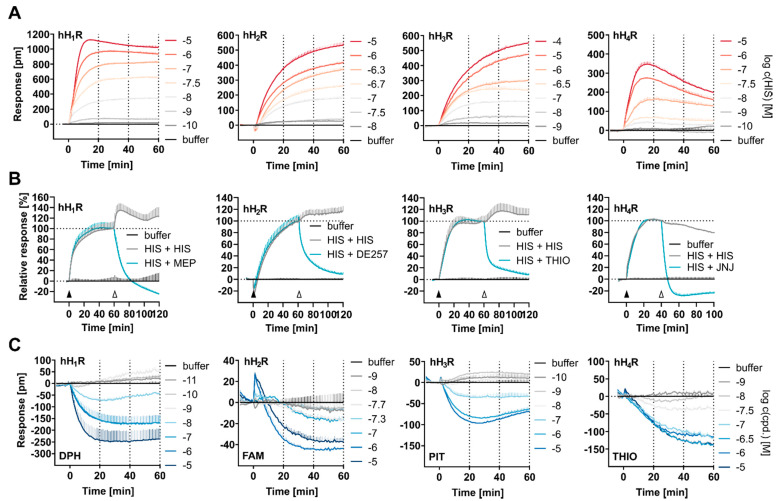
Implementation of the DMR assay for the hH_1–4_Rs stably expressed in HEK cells. (**A**) Representative DMR traces recorded with HEK hH_1–4_R cells upon stimulation with increasing concentrations of histamine. (**B**) The histamine induced DMR responses were reversible in HEK hH_1–4_R cells. The HEK hH_1–4_R cells were pre incubated with histamine at concentrations corresponding to the respective pEC_80_ value (hH_1_R = 316 nM, hH_2_R = 794 nM, hH_3_R = 1995 nM, hH_4_R = 501 nM, indicated by the filled arrow ▲) and the DMR response was recorded for 60 min (hH_1-3_R) or for 40 min (hH_4_R). Subsequently, a receptor specific antagonist was added (hH_1_R 10 µM MEP, hH_2_R 10 µM DE257, hH_3_R 10 µM THIO, hH_4_R 10 µM JNJ, empty arrow △) and the DMR was recorded for additional 60 min. (**C**) Constitutive activity was detected in HEK hH_1–4_R cells. Inverse agonism was observed at the hH_1_R for DPH, at the hH_2_R for FAM, at the hH_3_R for PIT, at the hH_4_R for THIO. Traces shown in (**A**–**C**) were corrected for the buffer and represent mean ± SEM of the technical triplicate. Traces shown in (**B**) were additionally normalized to the value recorded after 60 min (100%).

HEK hH_1–4_R cells were stimulated with increasing HIS concentrations and the DMR response was recorded for 60 min. Positively deflected and concentration dependent DMR traces were observed for all four HR subtypes (Figure 2A), where both the signal maximum and the time course varied depending on the HR subtype. Of note, no DMR response was detected in non-transfected HEK wildtype (wt) cells, neither for HIS nor for inverse agonists (Supplementary Figure S6A), demonstrating that the ligand induced DMR responses observed in HEK hH_1–4_R cells were HR mediated.

The highest amplitude and fastest increase in the DMR response was observed in HEK hH_1_R cells (1000 pm after 15 min; 10 µM HIS).

This kinetic profile of HIS induced DMR in HEK hH_1_R cells shows similarity to that observed in HeLa cells, which express the hH_1_R endogenously [71]. In HeLa cells, the positive DMR showed a peak response of approx. 300 pm within 3–5 min upon HIS addition, which decreased slightly and remained stable thereafter [71]. In A431 cells, which also express the hH_1_R endogenously, the positive DMR signal increased to a maximum value of approx. 500 pm within approx. 5 min after addition of HIS [72]. Afterwards, the DMR signal decreased steadily to the level of the baseline [72]. A similar kinetic profile was observed previously in our group using genetically engineered HEK293T-CRE-Luc-H_1_R-hMSR1 cells where the hH_1_R was co-expressed with the human macrophage scavenger receptor 1 (hMSR1), introduced to enhance the adhesion of HEK cells [20]. In HEK293T-CRE-Luc-H_1_R-hMSR1 cells, the positive DMR peaked at approx. 600 pm within 10 min after HIS addition and gradually decreased afterwards back to baseline [20,73]. These disparate kinetic profiles observed after stimulation of the hH_1_R with HIS were not surprising, as many characteristics of the different cell models used, e.g., receptor expression, expression patterns of (G) proteins, and/or cell adhesion, can affect the kinetic profile of the DMR response [73,74]. When comparing the kinetics of HIS in HEK hH_1_R cells with DMR traces of purinergic P2Y or muscarinic M3 receptors (both also Gα_q_ coupled and heterologously expressed in HEK cells) [44], no similarities were found.

Compared to HEK hH_1_R cells, the DMR response recorded for HEK hH_2_R and HEK hH_3_R cells were markedly different, showing no sharp maxima upon stimulation with HIS at a concentration of 10 or 100 µM within 60 min. Instead, the DMR signal increased slower, but steadily, reaching a highest amplitude ranging between 500–600 pm after 60 min. A unique feature of the hH_2_R mediated DMR response was a slight signal dip (Zoom-in in supplementary Figure S5) immediately after HIS addition, a phenomenon that was not observed within this study for any other HR subtype under the same experimental conditions. A signal dip was also observed for the Gα_s_ coupled GPCRs [29,37], e.g., EP2/4, which was stably expressed in HEK cells [37]. Ye Fang [29] explained such a signal dip by the fact that downstream signaling components involved in the signal transduction process are already compartmentalized and located at or near the cell membrane. Therefore, the recruitment of intracellular signal transduction components to activated receptors is less pronounced and other cellular signaling events are more salient leading to an initial decrease in local mass density [29]. However, one should be careful to interpret this as a reliable feature of Gα_s_ coupling.

Although the hH_2_R is reported as Gα_s_ coupled [57] and the hH_3_R is described as a Gα_i/o_ coupled receptor [57], the DMR traces recorded upon stimulation with HIS were similar in both signal amplitude and time course, except for the signal dip in the case of the hH_2_R (Figure 2A). This was surprising as we had expected that different G protein coupling would be associated with distinct DMR signaling profiles. Moreover, it was interesting that the signal amplitudes were similar because the expression level of the hH_3_R was approximately 20-fold lower compared to that of hH_2_R (Table 1), conflicting with the assumption that the signal amplitude is positively correlated with the level of receptor expression. Instead, it can be speculated that the receptor-specific signal transduction pathway plays a role.

Even though both the hH_3,4_Rs are structurally related and considered as Gα_i/o_ coupled receptors (Figure 1B), the recorded DMR traces of HEK hH_3_R and hH_4_R cells differed in both time course and signal amplitude (Figure 2A). Among the four human HR subtypes analyzed in this study, the lowest DMR response was recorded in HEK hH_4_R cells. The signal reached its maximum of approx. 300–400 pm within 10–20 min at the highest HIS concentration of 10 µM, and then declined continuously. Various Gα_i/o_ coupled receptors expressed in HEK cells (DP2 [41], CRTH2 [44]) or in CHO cells (NOP [75]) showed comparable kinetic profiles in DMR assays.

#### 2.2.2. Reversibility of HIS Induced DMR

To demonstrate the reversibility of the DMR responses, HEK hH_1–4_R cells were first treated with histamine at concentrations corresponding to the respective pEC_80_ value (hH_1_R = 316 nM, hH_2_R = 794 nM, hH_3_R = 1995 nM, hH_4_R = 501 nM; indicated by the filled arrow in Figure 2B) and the DMR was recorded for 60 min with HEK hH_1,2,3_R cells, or for 40 min with HEK hH_4_R cells. In the second step, a receptor-specific antagonist was added (10 µM mepyramine (MEP) for hH_1_R, 10 µM DE257 for hH_2_R, 100 µM thioperamide (THIO) for hH_3_R and 10 µM JNJ7777120 (JNJ) for hH_4_R; indicated by the empty arrow in Figure 2B; structures of antagonists are presented in supplementary Figure S1). As a control, HEK hH_1–4_R cells were also stimulated with HIS, but in the second step, instead of an antagonist, HIS was added at a concentration corresponding to the pEC_80_. This was to ensure that the observed effect was induced by the antagonist and not by the addition procedure disturbing the system. For all four HR subtypes, the HIS-induced signal was suppressed by addition of a receptor subtype-specific antagonist, and no decrease in the signal was observed in the controls, showing reversibility of the DMR signal.

#### 2.2.3. Constitutive Activity

Previously, all four HR subtypes have been reported as constitutively active in heterologous expression systems in canonical assays [23,76,77,78,79,80,81]. Constitutive (basal) activity describes the ability of GPCRs to produce a biological response in the absence of agonist binding by spontaneously adopting an active conformation [82]. Usually, the measurement of constitutive activity occurs by comparing the basal activity of a system comprising active-state receptors (e.g., transfected cells or high receptor expression) and without receptors (e.g., not transfected cell, low receptor expression) [83]. The basal activity should increase with increase in receptor expression. To assess the constitutive activity of HRs in the DMR assay we compared the DMR traces of the buffer controls (assay buffer w/o ligand) recorded for not transfected HEK cells with that recorded for HEK hH_1–4_R cells (Supplementary Figure S7). After an equilibration period of about 40 min, higher basal activity was measured in HEK hH_1,3,4_Rs compared to not transfected HEK wt cells. We interpret this as an indication that the receptors in this system are constitutively active. However, no difference in the basal activity was observed between HEK hH_2_R cells and HEK wt cells (Supplementary Figure S7). This may imply that the hH_2_R is either not constitutively active in this system, or that this activity is too weak to be detected in this system. To explore measurement of inverse agonism by DMR, HEK hH_1–4_R cells were stimulated with a receptor specific inverse agonist (hH_1_R: DPH, hH_2_R: FAM, hH_3_R: PIT, hH_4_R: THIO) at increasing concentrations. Constitutive activity, manifesting as negatively deflected DMR traces, was observed at all four receptor subtypes, differing in intensity depending on the HR-ligand combination. (Figure 2C). The weakest inverse activity was measured for the hH_2_R when stimulated with FAM. This implies that the hH_2_R is constitutively active, but much lower compared to hH_1,3,4_R. We previously anticipated this to be the case in view of supplementary Figure S7. We can rule out off-target effects for any HR-ligand combination, as none of the ligands elicited a DMR response in not transfected HEK wt cells (Supplementary Figure S6A).

#### 2.2.4. Assay Quality

For data analysis, the area under curve (AUC) was calculated for the DMR traces, which is a commonly applied concept for the assessment of dynamic pharmacological processes [38,73]. Compared to single point measurements, the integration over time provides a more accurate estimate of the overall response to a drug [84]. To assess assay quality, signal-to-background (S/B) ratios were estimated based on AUC over the entire measurement period of 60 min (AUC_60_) for both HIS and a respective inverse agonist using HEK hH_1–4_R cells. We are aware that the calculation of the S/B ratio using AUC appears problematic as the DMR signal does not represent an absolute measure, but rather a shift of the wavelength relative to the baseline. To alleviate this problem, we considered the stable baseline as the zero point and used the modulus of AUC for the estimation of the S/B ratio. This approximation is possible because the EnSpire software records and uses the last measuring point (repeat) in the baseline run as the calibration offset, which is subtracted from all repeats of the baseline and the final record (last repeat in the baseline was set to zero) [85].

**Figure 3:**
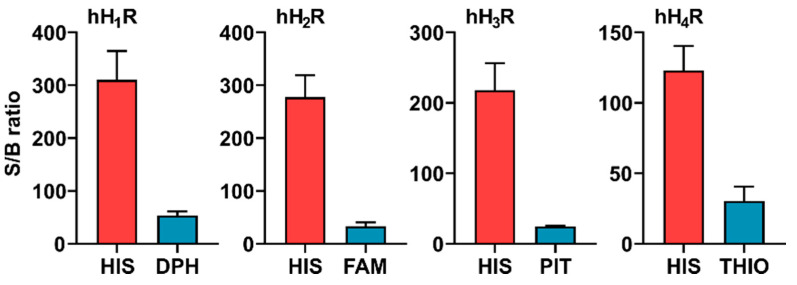
Signal-to-background (S/B) ratios estimated for HIS and inverse agonists using HEK hH_1–4_R cells. For the estimation of S/B values, the modulus of area under curve (AUC_60_) was calculated for the highest concentrations of HIS (10 µM in HEK hH_1,2,4_R cells and 100 µM in HEK hH_3_R cells) and the respective inverse agonist (10 µM DPH in HEK hH_1_R cells, 10 µM FAM in HEK hH_2_R cells, 10 µM PIT in HEK hH_3_R cells, 100 µM THIO in HEK hH_4_R cells), and divided by the respective AUC_60_ value determined for the buffer. For HIS, the positive AUC (above baseline) was used, whereas for the inverse agonists the negative AUC (below baseline) was calculated. The S/B ratios are presented as mean ± SEM from at least three independent experiments, each performed in triplicate.

For HIS, high S/B ratios were determined at the hH_1_R, hH_2_R, hH_3_R and hH_4_R, amounting to 308, 277, 218 and 123, respectively (Figure 3). Compared to HIS, the S/B ratios for the standard inverse agonists were markedly lower (S/B ratios: DPH (hH_1_R) = 53, FAM (hH_2_R) = 33, PIT (hH_3_R) = 25 and THIO (hH_4_R) = 30) as shown in Figure 3. In comparison, S/B ratios for HIS in [^35^S]GTPγS or miniG assays ranged from 2 to 30 [80]. Among other factors, high S/B ratios are beneficial for signal deconvolution studies, assessing efficacies and potencies of ligands, and investigating constitutive activities of receptors.

#### 2.2.5. Conversion of the DMR Responses to Concentration-Response-Curves (CRCs)

**Figure 4:**
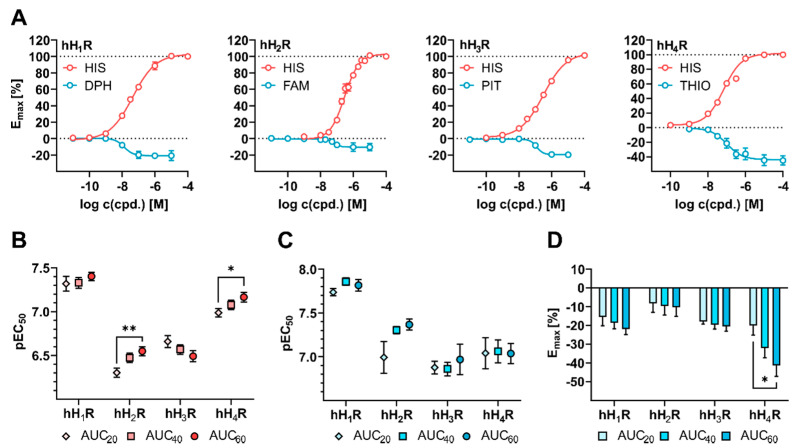
Analysis of DMR responses elicited by HIS (**red**) and inverse agonists (**blue**) in HEK hH_1–4_R cells. (**A**) CRCs determined for HIS and indicated inverse agonists at the hH_1–4_Rs using AUC_60_. The E_max_ values determined for the inverse agonists wee normalized to the highest histamine concentration applied for the respective receptor subtype. (**B**) pEC_50_ values for HIS resulting from CRCs constructed by using AUC_20_, AUC_40_ or AUC_60_ at the hH_1–4_Rs. (**C**) pEC_50_ values calculated for inverse agonists (DPH at the hH_1_R, FAM at the hH_2_R, PIT at the hH_3_R and THIO at the hH_4_R) resulting from CRCs constructed by using AUC_20_, AUC_40_ or AUC_60_ at the hH_1–4_Rs. (**D**) E_max_ values determined for DPH at the hH_1_R, FAM at the hH_2_R, PIT at the hH_3_R and THIO at the hH_4_R using the AUC_20_, AUC_40_ or AUC_60_. E_max_ values were normalized to the highest HIS concentration applied for the corresponding HR subtype. (**A**–**D**) All values are means ± SEM of at least three independent experiments, each performed in triplicate. Statistical difference relative to AUC_60_ was analyzed by one-way ANOVA followed by Dunnett’s multiple comparison test calculated as * *p* ≤ 0.05, ** *p* ≤ 0.01.

The optical traces (representations in Figure 2A, C) were converted to CRCs by calculating the AUC_60_ and plotting these values against the logarithmic concentrations of a compound (Figure 4A). The determined pEC_50_ and E_max_ values are summarized in Table 2. However, when calculating the S/B ratios, a slight dependency on the time interval used for the AUC calculations was observed (described in SM Text S1, Impact of the time interval used for calculations of AUC on S/B ratios, supplementary Figure S8 and supplementary Table S3). Moreover, a time-dependent potency of agonists was observed at the muscarinic M_3_ [86] and the neurotensin NTS_1_ [53] receptor in DMR assays. Therefore, we investigated whether the time interval used to calculate the AUC had an impact on the pEC_50_ and E_max_ values of HIS and the receptor specific inverse agonists.

For this purpose, additional CRCs were constructed using AUC calculations after 20 or 40 min (AUC_20, 40_) and compared to those from AUC_60_ (Supplementary Figure S9 and supplementary Table S4). For HEK hH_1_R cells, the time interval had no impact on the mean pEC_50_ values for HIS (Figure 4B). By contrast, in HEK hH_2_R and hH_4_R cells a significant increase in pEC_50_ values from AUC_20_ to AUC_60_ was observed for HIS (hH_2_R: from 6.30 ± 0.05 to 6.57 ± 0.05; hH_4_R: from 6.99 ± 0.05 to 7.15 ± 0.05, respectively), whereas in HEK hH_3_R a gradual decrease in mean pEC_50_ values was observed from AUC_20_ to AUC_60_ (from 6.66 ± 0.07 to 6.49 ± 0.06), which, however, was statistically not significant. For inverse agonists, the calculation of AUC after 20, 40 and 60 min had no significant impact on the mean pEC_50_ values (Figure 4C). Signal transduction of GPCRs involves a complex network of different spatially and temporally resolved events, each of which show individual kinetics and/or amplitudes [53,86,87]. As all this information is bundled in the DMR response, it was not surprising that the temporal component could have an impact on the pEC_50_ and E_max_ value depending on the specific signaling cascade triggered by the receptor ligand interaction. The E_max_ values gradually decreased from AUC_20_ to AUC_60_ for all four HR-inverse agonist combinations (Figure 4D; exact values in supplementary Table S4) but particularly for THIO at hH_4_R, where the mean E_max_ value showed a significant decrease from AUC_20_ to AUC_60_ (E_max_(AUC_20_) = −20.1 ± 5.0 to E_max_(AUC_60_) = −45.0 ± 5.7). The slow kinetics of the DMR response recorded for the inverse agonists can be considered as an explanation here (Figure 2A (HIS) versus Figure 2C (inverse agonists). In view of these results, the inclusion of the entire kinetic information (AUC_60_) appears preferable and was considered as the standard method to calculate pEC_50_ and E_max_ values in the following experiments.

#### 2.2.6. Functional Characterization of (Inverse) Agonists: Label-Free DMR versus Label-Dependent Techniques

There was a discrepancy between competition binding and DMR functional data determined for HIS using HEK hH_1,2_R cells (Table 2). The pK_i_ values for HIS in live cells were approximately 4 (hH_1_R) or 2 (hH_2_R) orders of magnitude lower compared to the pEC_50_ values in the DMR assay. Moreover, a discrepancy between affinity and potency was observed for pitolisant (PIT) at the hH_3_R, where the pK_i_ value was about 2 orders of magnitude larger compared to the pEC_50_ value in the DMR assay. As binding data reflect the strength of the receptor-ligand interaction, whereas functional responses are amplified translations of the receptor-ligand interaction, differences in this range are not uncommon and have been reported for example, for dopamine receptors [88]. We have previously stimulated HEK293T-CRE-Luc-hH_1_R-hMSR1 cells with HIS at increasing concentrations in the DMR assay [20]. The CRCs from AUC_40_ revealed a pEC_50_ value of 7.49 [20], which agrees with the result reported here (pEC_50_ = 7.38 ± 0.05).

To the best of our knowledge, we are the first to report functional DMR data for the hH_2–4_Rs, so no reference data was available. In order to compare the results, a miniG recruitment assay, recently implemented by Hoering et al. [80] for the entire HR family, was used. As the miniG recruitment assay was also performed with live HEK cells in real time, and the AUC used for data analysis, these results were particularly well suited as a reference. As a canonical alternative, a luciferase reporter gene assay was used. This assay was also performed with HEK cells but represents an endpoint measurement, in contrast to the kinetic measurements of DMR and miniG recruitment assays. Although the three assays measure different processes in the signal transduction cascade of HRs, in general, the pEC_50_ values were in good agreement, not differing more than one order of magnitude (Table 2). Exceptions are HIS at the hH_1_R (DMR vs. miniG) and HIS at the hH_3_R (DMR vs. luciferase). By contrast, higher discrepancies were observed regarding the efficacy of the inverse agonists. In general, inverse agonists were less efficacious in the miniG recruitment assay than in the DMR assay. However, as only one miniG protein-HR interaction was monitored rather than the holistic cellular response as in the DMR assay, this discrepancy is not surprising. A better agreement of E_max_ values was observed between the DMR and the luciferase reporter gene assay for THIO at the hH_4_R.

### 2.3. Dissecting HIS Induced DMR Signals in HEK hH_1–4_R Cells Using G Protein Modulators

#### 2.3.1. Impact of Individual Gα Protein Modulators on the DMR Response

**Figure 5:**
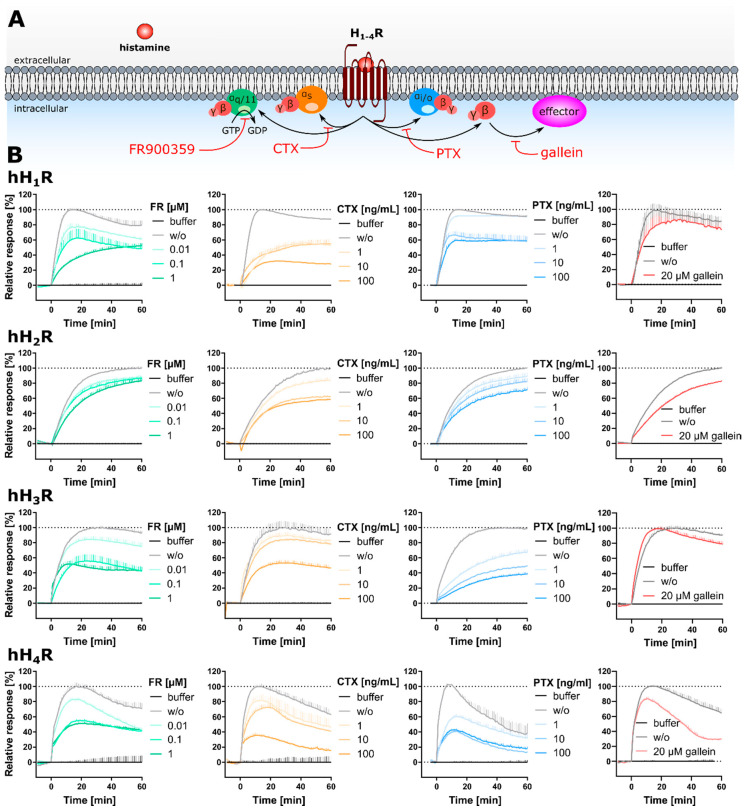
Effect of individual G protein modulators on the HIS induced DMR traces recorded in HEK hH_1–4_R cells. (**A**) Interference of the G protein modulators FR, CTX, PTX and gallein with histamine receptor mediated signaling. FR (alias UBO_−_QIC) selectively silences Gα_q/11_ signaling by blocking the GDP_−_release at concentrations 0.1 to 1.0 µM [18,41,44,45,46,47]. PTX selectively and irreversibly silences Gα_i/o_ at a concentration of 100 ng/mL by ADP_−_ribosylation at the Gα_−_subunit [20,33,37,40,41,42]. CTX locks the Gα_s_ protein in its GTP bound state by irreversible ADP-ribosylation leading to a permanent activation of the Gα_s_ protein, which is in turn uncoupled and no longer available for the GPCR [31,32,33,37,43] at a concentration of 100 ng/mL [31,32,37]. As this approach only masks the Gα_s_ protein coupled pathway the results should be interpreted with caution. Gallein (gal) is reported to reversibly bind to the Gβγ subunit (K_d_ = 422 nM) [92], preventing an interaction with effector proteins [92,93,94]. (**B**) Representative time courses of the HIS induced DMR response in HEK hH_1–4_R cells pre_−_treated with G protein modulator at the indicated concentrations overnight (PTX and CTX) or 30 min (FR and gallein) before measurement of stimulation with HIS (hH_1,2,4_R at 10 µM HIS, hH_3_R at 100 µM HIS). All traces were buffer-corrected and normalized to the maximum DMR response (wavelength shift in pm) of the untreated control (w/o). Data are presented as mean ± SEM of a technical triplicate.

As outlined above, depending on the HR subtype, different intensities and time courses of the DMR responses were observed when HEK hH_1–4_R cells were stimulated with HIS at increasing concentrations (Figure 2A). We investigated whether the receptor-specific DMR response was exclusively the result of an activation of the primary Gα protein dependent signaling pathway described in the literature (Figure 1B), or whether additional G proteins were involved in the HIS-induced DMR response. The contribution of endogenously expressed Gα proteins was analyzed using G protein pathway modulators FR900359 (FR), pertussis toxin (PTX), and cholera toxin (CTX; mechanisms for all three outlined in Figure 5A). CRCs were recorded for HIS in HEK hH_1–4_R cells in the absence and presence of CTX, PTX (both at concentrations of 1.00, 10.0 and 100 ng/mL) and FR (at concentrations of 0.01, 0.10 and 1.00 µM). In every experiment, HEK hH_1–4_R cells stimulated with HIS without (w/o) modulators served as 100% control. DMR traces recorded at the highest histamine concentration in the absence and presence of the respective modulator were compared (Figure 5B) and, as before, AUC_60_ CRCs were constructed (Supplementary Figure S10). The corresponding E_max_ and pEC_50_ values are summarized in supplementary Figure S11.

- hH_1_R

**Figure 6:**
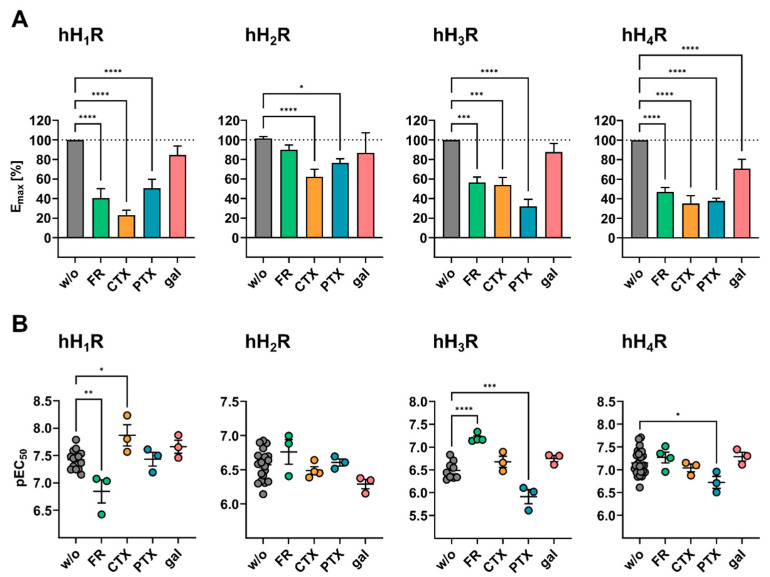
Effect of individual G protein modulators on the efficacy and potency of HIS at hH_1–4_Rs. E_max_ and pEC_50_ values were determined for HIS in the absence and the presence of G protein modulators. (**A**) Bar chart of E_max_ values determined for HIS in absence (w/o, grey) and presence of FR (green, 1 µM), CTX (orange, 100 ng/mL), PTX (blue, 100 ng/mL) and gal (red, 20 µM). The E_max_ values were calculated using AUC_60_ at the highest HIS concentration (10 µM for hH_1,2,4_R and 100 µM for hH_3_R) and normalized to the AUC_60_ of the untreated control (100%) and buffer (0%) values. (**B**) Scatter plot of the pEC_50_ values in absence (grey) and presence of G protein modulators at the concentration stated above. The pEC_50_ were determined by plotting the AUC_60_ against the respective HIS concentration. (**A**,**B**) Data presented are means ± SEM of at least three independent experiments, each performed in triplicate. Statistical difference relative to the control was analyzed by one-way ANOVA followed by Dunnett’s multiple comparison test. Significance levels are indicated by asterisks (* *p* ≤ 0.05, ** *p* ≤ 0.01, *** *p* ≤ 0.001, **** *p* ≤ 0.0001).

Because the Gα_q/11_ pathway is considered canonical for the hH_1_R [56,57], a strong decline of the DMR response was expected upon incubating the HEK hH_1_R cells with the Gα_q/11_ modulator FR. When HEK hH_1_R cells were treated with 1.00 µM FR, the time course of the DMR signal for HIS was noticeably altered, but not with 0.01 µM or 0.1 µM FR (5B green traces). In the former case, no maximum was observed and the DMR response was slower. However, even the highest FR concentration of 1.00 µM was not sufficient to eradicate the HIS DMR response (Figure 5B, green traces), although the E_max_ value was reduced to 41 ± 9.5% (Figure 6A). Likewise, Lieb et al. were also not able to completely suppress the HIS induced DMR in HEK293T-CRE-Luc-hH_1_R-hMSR1 cells in the presence of 1.00 or 10.0 µM FR [20]. For comparison, a concentration of 1.00 µM FR was enough to completely disrupt the DMR response of the muscarinic M_3_R, which solely couples to Gα_q/11_ [44]. Thus, we conclude that the failure to completely suppress the DMR signal was not due to insufficient FR concentration, but rather that the residual signal in HEK hH_1_R cells comes from additional (G) protein interactions. The significantly reduced pEC_50_ in the presence of 1.00 µM FR (6.81 ± 0.15) could be caused by inactivation of the Gα_q/11_ protein abolishing the Gα_q/11_ positive modulation, an effect seen when the G protein stabilizes the active conformation of the receptor [24,90].

Surprisingly, masking of the Gα_s_ signaling pathway with CTX had a greater effect on the DMR response of the hH_1_R (Figure 5B, orange traces) than FR. Even the lowest concentration of 1.00 ng/mL CTX enormously altered both the maximum amplitude, and the time course of the HIS induced DMR response. In this case, the DMR response was slowed down and showed no signal maximum as observed for untreated HEK hH_1_R cells. An increase in CTX concentration to 100 ng/mL further reduced the signal amplitude and led to a deceleration of the DMR signal. Unexpectedly, among the investigated modulators, 100 ng/mL CTX had the strongest effect on E_max_ at the hH_1_R lowering the value to 23 ± 4.9% (Figure 6A), suggesting that the Gα_s_ protein is involved in the hH_1_R mediated DMR signal. Indeed, it has been shown that the hH_1_R can functionally interact with the Gα_s_ protein in HEK cells overexpressing both the receptor and the Gα_s_ protein [60,91]. The inhibition of Gα_s_ pathway led to a significant increase in the pEC_50_ value (7.87 ± 0.19; Figure 6B). It is possible that the uncoupling of Gα_s_ may have enhanced Gα_q_ protein interaction with the hH_1_R, or Gα_s_ may even act as a negative modulator at hH_1_R. Further investigations are necessary to determine the mechanism involved.

The inhibition of Gα_i/o_ signaling pathway with PTX reached its maximum effect at a concentration of 10.0 ng/mL at the hH_1_R (Figure 5B, blue traces). Except for decreasing the signal amplitude to maximum 50 ± 9.3% of E_max_ relative to control cells (Figure 6A), PTX had no effect on the time course of the DMR signal, suggesting Gα_i/o_ protein involvement in hH_1_R signal transduction. This is in good accordance with the literature [20,59,61]. For example, Lieb et al. showed that the hH_1_R also signals via Gα_i/o_ in the DMR assay using HEK293T-CRE-Luc-hH_1_R-hMSR1, as the DMR signal was completely abolished by 100 ng/mL PTX [20].

- hH_2_R

Pretreatment of HEK hH_2_R cells with increasing CTX concentrations led to a gradual decrease in the signal amplitude relative to the untreated control, but, in contrast to HEK hH_1_R cells, did not alter the shape of the DMR time course (Figure 5B, orange traces). At 100 ng/mL CTX, 62 ± 7.7% of the hH_2_R signal was retained; a significant effect, but not as pronounced as with the other three HR subtypes (Figure 6A; hH_1_R 23 ± 4.9%, hH_3_R 54 ± 7.6% and hH_4_R 35 ± 7.9% signal retention). This was unexpected, as the hH_2_R is commonly considered as a Gα_s_-coupled receptor [56,95]. Furthermore, 100 ng/mL CTX have been shown to almost completely abolish the agonist induced DMR response of the Gα_s_-sensitive β_2_ adrenoreceptor (β_2_R) expressed by different cell types endogenously or heterologously [31,32,37]. Moreover, the pEC_50_ value of HIS remained unaffected by the treatment with CTX (Figure 6B). We expected that uncoupling of the Gα_s_ protein with CTX would negatively affect the pEC_50_ value of HIS, as was the case with hH_1_R after the Gα_q/11_ protein was inactivated by FR. These data suggest that additional signaling pathways contribute to the DMR response in HEK hH_2_R cells.

Apart from Gα_s_, it is known that the Gα_q/11_ protein can play a considerable role in H_2_R signal transduction, dependent on the cellular background [56]. This was not confirmed in the DMR assay as the Gα_q/11_ modulator FR was almost completely ineffective, even at a concentration of 1.00 µM (Figure 5B green traces). Although a stepwise decline of the DMR response was observed with increasing PTX concentrations to investigate the involvement of Gα_i/o_ in the HIS induced DMR (Figure 5B blue traces), the effect was less pronounced than with CTX (E_max_ = 77 ± 4.2% at 100 ng/mL PTX versus E_max_ = 62 ± 7.7%; Figure 6A). Strikingly, in contrast to the other three HR subtypes, the individual modulators FR, CTX and PTX, had little effect on the HIS induced DMR response in HEK hH_2_R cells. Two explanations can be considered. Firstly, silencing of one pathway may have caused the hH_2_R to switch to other pathways, indicating promiscuous signal transduction of the receptor. Secondly, these results may also indicate the involvement of other effectors, e.g., Gα_z_ or Gα_12/13_ [59], in the hH_2_R mediated DMR response.

- hH_3_R

As expected, inhibition of the Gα_i/o_ signaling pathway with PTX in HEK hH_3_R cells had a dramatic impact on the DMR response to 100 µM HIS, for both the E_max_ and pEC_50_ values. Even 1.00 ng/mL of PTX was sufficient to decelerate the hH_3_R DMR response (Figure 5A, blue traces) and to reduce the E_max_ to 63 ± 14% (Figure 6A), roughly a 4× more reduction than for hH_1,2_Rs. However, we failed to completely suppress the signal, as at 100 ng/mL PTX 32 ± 7.2% of E_max_ remained. By contrast, Shi et al. described that the HIS response was disrupted by 100 ng/mL of PTX in a CRE-driven luciferase activity assay using HEK cells stably expressing the hH_3_R [42]. Moreover, for other Gα_i/o_ coupled receptors, e.g., the muscarinic M_2_ [48], or prostaglandin CRTH_2_ [37], PTX at a concentration of 100 ng/mL was sufficient to completely disrupt the DMR signal in CHO or HEK cells. Thus, we expect that 100 ng/mL PTX was sufficient to inactivate Gα_i/o_ mediated signaling and conclude that other (G) proteins were involved in the hH_3_R mediated DMR response. The pEC_50_ values declined with increasing PTX concentrations from 6.49 ± 0.06 (control) to 5.75 ± 0.17 and 5.91 ± 0.15 (10.0 and 100 ng/mL of PTX, respectively; Figure 6B). As described above, a similar phenomenon was observed for the hH_1_R when its canonical Gα_q/11_ signaling pathway was blocked with 1 µM FR. We believe the same hypothesis to be true here, namely that the Gα_i/o_ protein stabilizes an active conformation of the hH_3_R, resulting in decreased pEC_50_ values when blocked. Consistent with literature [57], this suggests that the Gα_i/o_ protein plays a major role in hH_3_R mediated signal transduction. However, as it was not possible to completely abrogate the DMR response with PTX, other G protein (in)dependent signaling pathways might be involved as well.

The Gα_q/11_ modulator FR at increasing concentrations had no effect on the time course of the HIS induced DMR response, but did decrease the signal amplitude (Figure 5B, green traces). A decrease in E_max_ to about 95 ± 5.3% was observed in the presence of 0.01 µM FR, whereas 0.10 µM FR significantly reduced the E_max_ value to 60 ± 6.4%. A ten-fold increase in FR concentration to 1.00 µM decreased the E_max_ by only additional 3% compared with 0.10 µM FR, indicating that at the latter concentration of FR the Gα_q/11_ dependent DMR was almost completely inhibited in HEK hH_3_R cells (Figure 6A). Strikingly, the pEC_50_ value was significantly increased to 7.20 ± 0.05 after treatment with 1.00 µM FR referring to the pEC_50_ of 6.49 ± 0.06 in control cells (Figure 6B). We did not expect this impact of Gα_q/11_ inhibition because hitherto the hH_3_R has been described as a Gα_i/o_ selective receptor and to date, no evidence has been provided that the hH_3_R is capable of activating a Gα protein other than Gα_i/o_ [91].

Masking the Gα_s_ signaling with CTX did not affect the time course of the HIS-induced hH_3_R mediated DMR response elicited by HIS, but again the signal amplitude was affected (Figure 5B). The E_max_ values decreased to 77 ± 9.0% or 76 ± 6.3% after treatment with 1.00 or 10.0 ng/mL of CTX, respectively and was significantly reduced to 54 ± 7.61% in the presence of 100 ng/mL CTX compared to control cells (Figure 6A). In comparison, the E_max_ value at the hH_1_R was already reduced to 43 ± 9.5% at a concentration of 1 ng/mL of CTX. Therefore, we reason that Gα_s_ is not as involved in signal transduction at the hH_3_R as at the hH_1_R. This assumption was further supported by the fact that the pEC_50_ value was not significantly affected by the treatment with CTX (Figure 6B).

- hH_4_R

Pre-incubation of HEK hH_4_R cells with PTX at increasing concentrations to block the Gα_i/o_ protein had no influence on the time course but did affect the signal amplitude of the HIS induced DMR response (Figure 5B). Similar to the hH_3_R response, even at 1 ng/mL PTX the E_max_ was lowered to 51 ± 6.8% (Figure 6A). However, we failed to completely displace the HIS induced DMR response at the hH_4_R by PTX, even at a concentration of 100 ng/mL (Figure 6A; E_max_ = 38 ± 2.9%). Elsewhere, in a luciferase reporter gene assay with HEK293-EBNA cells transfected with the hH_4_R (referred to as GPRv53), 100 ng/mL PTX completely abolished the HIS induced response [96]. However, unlike the hH_3_R response, an increase in PTX concentration had no effect on pEC_50_ values in HEK hH_4_R cells (Figure 6B).

The contribution of the Gα_s_ protein was analyzed by pre-treating the cells with CTX at increasing concentrations. Figure 5B (hH_4_R orange traces) shows that the maximum responses declined stepwise, whereas time courses of the HIS induced DMR remained unaltered (Figure 5B). Only at the highest CTX concentration of 100 ng/mL, did the signal decrease substantially (E_max_ = 35 ± 7.9%; Figure 6A). Analogous to the hH_3_R, we assume that Gα_s_ is of smaller importance in the signal transduction of the hH_4_R compared to the hH_1_R, where the E_max_ value was reduced to 43 ± 9.5% with 1 ng/mL CTX. Moreover, the pEC_50_ value for HIS in HEK hH_4_R cells was not affected in the presence of CTX (Figure 6B). The Gα_q/11_ modulator FR at increasing concentrations led to a stepwise decrease in the hH_4_R mediated DMR response. FR at a concentration of 0.10 µM was sufficient to decrease the DMR signal to 71 ± 18% relative to the untreated control, and a further decline was observed in the presence of 1.00 µM FR (E_max_ = 47 ± 4.5%; Figure 6A). Different to the hH_3_R, the pEC_50_ value was unaltered by the blockage of the Gα_q/11_ protein with FR (Figure 6B).

#### 2.3.2. Impact of the Gβγ Protein Modulator Gallein on the DMR Response upon Stimulation with Histamine

In addition to Gα, the Gβγ dimer is also able to interact with effectors in the signal transduction process. A contribution of Gβγ to the DMR response was assessed by means of the small modulatory molecule gallein [92,93,94]. Pretreatment of HEK hH_1–3_R cells with 20 µM gallein prior to stimulation with HIS led only to a marginal reduction of the E_max_ value to approximately 85% compared to control cells (Figure 5B, red traces and Figure 6A). In the case of the hH_4_R, the same gallein concentration significantly reduced the E_max_ value to 71 ± 9.3%. The pEC_50_ value for HIS remained unaltered by the treatment with gallein (Figure 6B). The modulatory effect of gallein on E_max_ values was markedly weaker at hH_1–4_Rs than for individual Gα modulators (1.00 µM FR, 100 ng/mL CTX and 100 ng/mL PTX). This may indicate that the endogenous Gβγ subunit plays a minor role in hH_1–4_Rs signal transduction in the DMR assay. Previous investigations using the cAMP-sensitive luciferase reporter gene assay with hH_1,2_Rs stably expressed in HEK293T cells also showed gallein as ineffective at reducing signal response (hH_1_R [20] or hH_2_R [97]). Unfortunately, to the best of our knowledge, comparable investigations with gallein concerning hH_3,4_Rs expressed in HEK cells were not available. Lavenus et al. [45] came to a similar conclusion when investigating the effect of 20 µM gallein on the Angiotensin II-induced response in HEK293-AT_1_R cells using the label-free surface plasmon resonance (SPR) technique. Further experiments are therefore necessary to clarify the involvement of Gβγ dimer in the signal transduction mediated by the hH_1–4_Rs.

#### 2.3.3. Impact of Gα Protein Modulator Combinations on the Histamine Induced DMR Response

**Figure 7:**
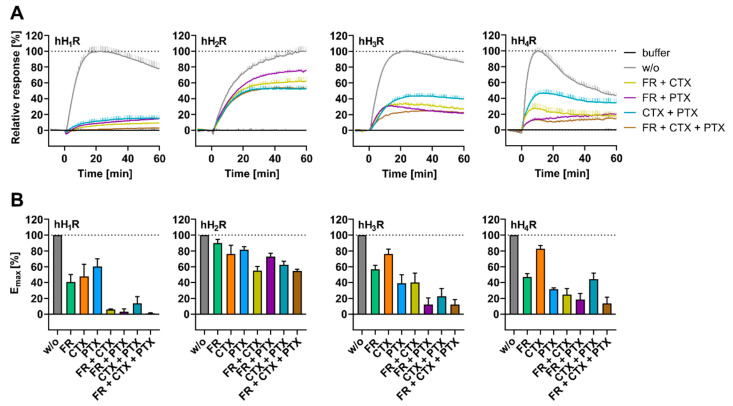
Impact of a combined application of Gα protein modulators FR, CTX and PTX on the DMR response at hH_1–4_R. (**A**) Representative DMR traces recorded for HIS in the absence (w/o) and in the presence of Gα protein modulators FR (1 µM, 30 min before measurement), CTX and/or PTX (both 10 ng/mL overnight) in HEK hH_1–4_R cells. (**B**) The AUC_60_ was calculated for the traces and normalized to the AUC_60_ of the untreated control (10 µM HIS without (w/o) modulator (100%) and to the AUC_60_ of the buffer control (0%). The values represent mean ± SEM of three independent experiments each performed in triplicate.

None of the four HR subtypes displayed completely suppressed DMR signals with single G protein modulators (Figure 6A). These results prompted us to investigate whether a complete inhibition of the DMR signal in HEK hH_1–4_R cells is achievable by combining the Gα protein modulators PTX, CTX and FR. At this point it should be noted that PTX and CTX were used at a concentration of 10.0 ng/mL instead of 100 ng/mL to avoid off target effects, which was usually sufficient to achieve the maximum effect (Supplementary Figure S11). HEK hH_1–4_R cells were treated with indicated modulators prior to stimulation with 10 µM HIS (Figure 7).

In HEK hH_1_R cells, each of the modulator combinations changed the time course of the HIS induced DMR response (Figure 7A), consistent with results from experiments with individual modulators (Figure 5B). Substantial depression of E_max_ to 14 ± 8.4% was seen in HEK hH_1_R cells after pretreatment with a combination of PTX and CTX (Figure 7B), corroborating with our previous results for the individual contributions of Gα_s/i/o_. A stronger reduction of the signal was observed when combining either PTX or CTX with FR, where the signal was reduced almost to the basal level (E_max_(PTX + FR) = 3.4 ± 3.5%, E_max_(CTX + FR) = 5.7 ± 0.8%; Figure 7B). The DMR signal was completely removed with a combination of the three modulators (PTX, CTX and FR). Therefore, we hypothesize that the HIS induced DMR response observed in HEK hH_1_R cells were exclusively transmitted via the three main classes of G proteins, namely Gα_q/11_, Gα_s_ and Gα_i/o_ proteins.

In accordance with the observations on individually applied Gα protein modulators (Figure 6), none of the modulator combinations altered the time course of the DMR response in HEK hH_2_R cells (Figure 7A). Surprisingly, the HIS induced DMR response in HEK hH_2_R cells was not even reduced by half upon treatment with a triple modulator combination (E_max_ (CTX, PTX, FR) = 55 ± 2.2%). In supplementary Figure S11, we showed that for both PTX and CTX, increasing the concentration from 10 ng/mL to 100 ng/mL no longer significantly reduced the DMR signal at the hH_2_R and FR had no effect on the E_max_ value at hH_2_R. Thus, we can exclude that PTX and CTX at a concentration of 10 ng/mL might not have been sufficient to completely inhibit the respective signaling pathways. Beyond this, at the hH_1,3,4_Rs, the same modulator combination caused a more pronounced decrease in the E_max_ value (Figure 7). Both arguments suggest that the weak impact of the triple modulator combination on the E_max_ was a hH_2_R-specific phenomenon. We conclude that in HEK hH_2_R cells the Gα_q/11_, Gα_s_ and Gα_i/o_ are not mainly responsible for the HIS induced DMR response, opposed to the hH_1,3,4_Rs. Referring to the aforementioned hypotheses constructed from the individually applied modulators, it appears that the hH_2_R is not only promiscuous with these Gα proteins, but there is also growing evidence for a possible interaction of hH_2_R with the Gα_12/13_ and/or Gα_z_, which are endogenously expressed in HEK cells [62].

In experiments with individually applied Gα modulators, a marked deceleration of the DMR response was observed in HEK hH_3_R cells in the presence of 10 ng/mL PTX (Figure 5B). As expected, such a retardation of the DMR signal was observed when HEK hH_3_R cells were pre-treated with modulator combinations comprising 10 ng/mL of PTX (Figure 7A). Unexpectedly, a combination of 1 µM FR + 10 ng/mL of CTX decelerated the DMR response. Moreover, the same combination (FR + CTX) markedly reduced the E_max_ to 40 ± 12% (Figure 7B). Both the impact on the time courses and the reduced E_max_ value in the presence of FR + CTX suggest that Gα_q/11_ and Gα_s_ are involved in the hH_3_R mediated DMR response. However, in comparison, a stronger decrease in E_max_ value was observed when combining 1 µM FR with 10 ng/mL of PTX to jointly inhibit Gα_q/11_ and Gα_i/o_ signaling pathways. This modulator combination reduced the E_max_ to 12 ± 8.3%, reaching a plateau that was found to be non-suppressible by the triple modulator combination of FR + CTX + PTX (E_max_ = 12 ± 6.4%; Figure 7B) suggesting that Gα_i/o_ and Gα_q/11_ played a more pronounced role in the hH_3_R mediated DMR response to HIS than Gα_s_. Again, as with the hH_2_R, the HIS induced DMR response was not completely ablated by the triple modulator combination. Inter alia, one possible explanation for this might be an involvement of additional G proteins such as Gα_12/13_ and/or Gα_z_. However, it should be noted that, unlike for the hH_1,2_Rs, the concentration of CTX in the triple modulator combination (FR + CTX + PTX) is a factor to be considered. In experiments with CTX alone (Supplementary Figure S11), 10 ng/mL CTX were not sufficient to achieve the maximum effect. Precisely, in the presence of 10 ng/mL an E_max_ value of 76 ± 6.3% was obtained, whereas 100 ng/mL CTX reduced the E_max_ value to 54 ± 7.6%. Although this difference was not determined to be significant (one-way ANOVA analysis followed by Tukey’s multiple comparison test; *p* = 0.1980), we still find it worth mentioning.

Likewise, we examined the influence of modulator combinations on the DMR signal in HEK hH_4_R cells. We would like to note that the differences in E_max_ values between the different modulator combinations were subtly nuanced rather than clear, just as with the individual modulators in Section 2.3.1. None of the Gα modulators effected the time courses of the HIS induced DMR responses when applied individually (Figure 5B). However, in combination, FR + PTX and FR + CTX + PTX altered the time course of the DMR response to HIS (Figure 7A). In both cases the DMR signal showed no peak and did not decline continuously, as observed in control experiments without modulators (Figure 2A). Instead, the DMR response increased steadily over time upon stimulation with HIS (Figure 7A). We took this as a hint that Gα_q/11_ and Gα_i/o_ have more impact on the signal transduction of hH_4_R in HEK cells than Gα_s_; nevertheless, the involvement of the latter should not be neglected. This opinion was enforced when E_max_ values were considered (Figure 7B); the treatment of HEK hH_4_R cells with CTX + PTX decreased the E_max_ to 44 ± 7.7%, whereas addition of FR (FR + CTX + PTX) reduced the E_max_ to a final value of 14 ± 8.0%, relative to control cells. It is also remarkable that a jointly inhibition of Gα_q/11_ and Gα_i/o_ signaling pathways with FR + PTX decreased the maximum response by almost the same level (E_max_ = 19 ± 7.7%) as the triple combination FR + CTX + PTX. Unexpectedly, the E_max_ value in presence of 10 ng/mL CTX + 10 ng/mL PTX was higher (E_max_ = 44 ± 7.7%) than that upon treatment with 10 ng/mL of PTX alone (E_max_ = 32 ± 1.9%). This might be due to the mechanism of action of CTX, as CTX does not directly inhibit the Gα_s_ protein, but rather masks the Gα_s_ dependent signaling pathway by permanent Gα_s_ protein activation. Similar to the hH_3_R, the inhibition of the three signaling pathways was not sufficient to completely remove the hH_4_R mediated response, as 14 ± 8.0% of E_max_ remained after treatment with FR + CTX + PTX (Figure 7B). Again, as with the hH_3_R, this demonstrates that signal transduction of the hH_4_R overexpressed in HEK cells occurred mainly through activation of Gα_i/o_, Gα_s_ and Gα_q/11_ proteins, but the DMR signal might also arise from either Gβγ, Gα_12/13_ and/or Gα_Z_ proteins.

Of note, the two structurally related receptor subtypes hH_3_R and hH_4_R have similar coupling specificities to Gα proteins, and so it is unsurprising that so that inhibition of the corresponding Gα signaling pathways led to comparable reduction in E_max_ values.

### 2.4. Investigation of HIS Induced DMR Signaling in G Protein Knock Out Cells

#### 2.4.1. Expression of hH_1–4_Rs in ΔGα_s_ HEK Cells

In addition to the concept of classical pharmacology, namely the employment of specific G protein modulators as molecular tools to elucidate cellular processes, we explored a molecular biology approach to better understand the contribution of individual Gα isoforms to the hH_1–4_R mediated DMR response using CRISPR/Cas9 modified HEK cells devoid of distinct Gα proteins (ΔGα_x_ HEK cells). For the generation of ΔGα_x_ HEK hH_1–4_R cells the ΔGα_s/l_ HEK [63], ΔGα_q/11_ HEK [44], ΔGα_12/13_ HEK [64] and ΔGα_six_ HEK [41] cells were stably transfected with the pIRESneo3-SP-FLAG-hH_1–4_R constructs and used as polyclonal cell lines. We confirmed the expression of the hH_1–4_Rs in ΔGα_x_ HEK hH_1–4_R cells by radioligand saturation binding using live cells (Supplementary Figure S3). The expression levels of hH_1–4_Rs in ΔGα_x_ HEK hH_1–4_R cells were calculated as mentioned for HEK hH_1–4_R cells (Table 1). When comparing the expression levels of respective HR subtypes in HEK hH_1–4_R cells with those in ΔGα_x_ HEK hH_1–4_R cells (e.g., expression of the hH_1_R in HEK hH_1_R versus in ΔGα_x_ HEK hH_1_R cells), we noted that the expression levels of hH_2–4_Rs were in the same range. In the case of the hH_1_R, the expression level was determined to be 10-fold lower in ΔGα_s/l, q/11, 12/13_ HEK hH_1_R cells compared to HEK hH_1_R cells. On the one hand, this difference may be due to the single clone selection procedure by which only the highest response HEK hH_1_R cells were obtained, thereby having the highest receptor expression level. The fact that only the binding capacity but not the affinity of the radioligand [^3^H]MEP was affected would argue in favor of this. However, this is contradicted by the fact that no difference in expression level was observed between the single clone HEK hH_2–4_R and polyclonal ΔGα_x_ HEK hH_2–4_R cells. On the other hand, we cannot exclude that knock-out of Gα proteins might have impaired either the expression of the hH_1_R or the detection of the binding capacity of the radioligand [^3^H]MEP to the hH_1_R in ΔGα_x_ HEK hH_1_R cells. This suspicion arose when we failed to detect the expression of the hH_1_R in ΔGα_six_ hH_1_R cells by radioligand saturation binding (Supplementary Figure S3), although a concentration-dependent signal was detected in the DMR assay when these cells were stimulated with HIS (Section 2.4.2 and supplementary Figure S12). By contrast, no DMR signal was observed in ΔGα_six_ HEK cells devoid of the hH_1_R (Supplementary Figure S6), suggesting that the hH_1_R was expressed in ΔGα_six_ HEK hH_1_R cells. For MEP, which has been reclassified as an inverse agonist [98], multiple binding sites differing in affinity and binding capacity for the H_1_R have been reported [99,100]. Moreover, the intrinsic negative efficacy of MEP is thought to be due to the stabilization of a G-protein-coupled state of the H_1_R that is not capable of eliciting a response [100]. Considering this, we argue in favor of the latter hypothesis, namely that the absence of Gα proteins may have affected the binding of [^3^H]MEP to the hH_1_R. However, as this research project is focused on the results in the DMR assay, we have not pursued this issue. The pK_d_ values determined with both HEK hH_1–4_R and ΔGα_x_ HEK hH_1–4_R cells were in very good agreement with literature data (Table 1), except for ΔGα_12/13_ hH_2_R and ΔGα_six_ hH_2_R. In both cases, the pK_d_ value increased significantly to 7.98 + 0.05 (ΔGα_12/13_ hH_2_R, *p* < 0.0001) and 7.86 + 0.06 (ΔGα_six_ hH_2_R, *p* = 0.0004) relative to the value determined using HEK hH_2_R cells (pK_d_ = 7.19 + 0.06). Apparently, the absence of Gα_12/13_ facilitates the binding of the radioligand [^3^H]DE-257 to the hH_2_R.

**Figure 8:**
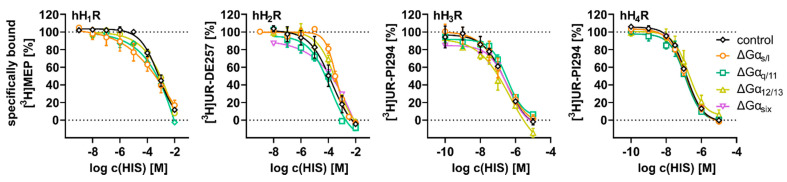
Radioligand displacement curves determined for histamine (HIS) at HEK hH_1__−4_R and ΔGα_x_ hH_1__−4_R cells. HIS was incubated at indicated concentrations in the presence of 5 nM [^3^H]mepyramine ([^3^H]MEP) at the hH_1_R, 50 nM [^3^H]UR_−_DE257 at the hH_2_R, 2 nM or 5 nM [^3^H]UR_−_PI294 hH_3_R or hH_4_R, respectively. The non_−_specific binding was determined in the presence of DPH (hH_1_R), FAM (hH_2_R) or HIS (hH_3,4_Rs), each at a final concentration of 10 µM. The non_−_specific binding was subtracted from the total binding to receive the specific binding. Specific binding was normalized to the buffer value (100%) and the corrected non_−_specific binding value (0%). Each point represents mean ± SEM of at least three independent experiments, each performed in triplicate.

The impact of Gα protein knock-out on the affinity of HIS to the hH_1–4_Rs was analyzed by radioligand competition binding with HIS as a competitor using live ΔGα_x_ HEK hH_1–4_R cells (Figure 8 and supplementary Table S2). Of note, such experiments were not performed with ΔGα_six_ HEK hH_1_R, as the expression of hH_1_R was not detectable in saturation binding experiments. Unfortunately, in ΔGα_s/l, q/11_ HEK hH_1_R cells a pK_i_ value could not be determined for HIS due to an ambiguous curve fit of the data. Although not statistically significant (*p* = 0.0545, *t*-test two-tailed), the pK_i_ value for HIS in ΔGα_12/13_ HEK hH_1_R cells (pK_i_ = 2.23 ± 0.37) was approx. one order of magnitude lower than at HEK hH_1_R cells (pK_i_ = 3.37 ± 0.29). While the absence of Gα_s/l_ or Gα_q/11_ proteins in ΔGα_s/l, q/11_ HEK hH_2_R cells had no impact on the pK_i_ of HIS (pK_i_ = 3.68 ± 0.09 and 4.23 ± 0.11, respectively), the value decreased approx. two-fold at ΔGα_six_ HEK hH_2_R cells (pK_i_ = 1.82 ± 0.28) compared to HEK hH_2_R cells (pK_i_ = 4.32 ± 0.38). The listed discrepancy of the pK_i_ values of HIS at the hH_1,2_Rs were not surprising, as saturation binding experiments (Table 1) demonstrate that the absence of Gα proteins can positively or negatively impact ligand binding at the hH_1,2_Rs. The pK_i_ values determined for HIS using ΔGα_x_ HEK hH_3,4_R cells were in good agreement with literature data and the results determined with HEK hH_3,4_R cells (Supplementary Table S2).

#### 2.4.2. Stimulation of ΔGα_x_ HEK hH_1–4_R Cells in the DMR Assay with HIS

**Figure 9:**
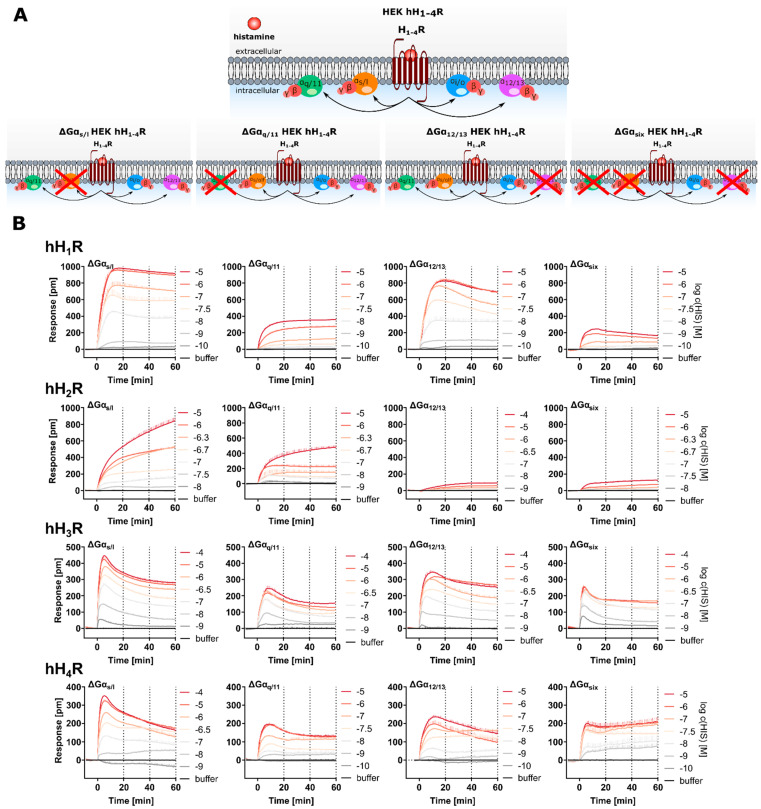
DMR responses recorded in Gα protein knock_−_out HEK hH_1__−__4_R cells upon stimulation with HIS. (**A**) Schematics of used Gα protein knock_−_out HEK (ΔGα_x_ HEK) cells. The ΔGα_x_ HEK cells lacking either the Gα_s/l_ (ΔGα_s/l_ HEK) [63], the Gα_q/11_ (ΔGα_q/11_ HEK) [44], the Gα_12/13_ (ΔGα_12/13_ HEK) [64] or six Gα proteins (ΔGα_s/l, q/11, 12/13_ = ΔGα_six_ HEK) [41] were stably transfected with hH_1__−__4_Rs. The knocked_−_out Gα protein is marked with a red “X”. HEK hH_1__−__4_R cells, expressing all four G protein classes were used as reference. (**B**) The ΔGα_x_ HEK hH_1–4_R cells either lacking the Gα_s/l_ (ΔGα_s/l_), Gα_q/11_ (ΔGα_q/11_), Gα_12/13_ (ΔGα_12/13_) or Gα_s/l_, _q/11_, _12/13_ (ΔGα_six_) proteins were stimulated with indicated HIS concentration and the DMR response was recorded for 60 min. Depicted are representative DMR traces, which were corrected for the buffer. Each trace represent mean ± SEM of a representative experiment performed in triplicate.

**Figure 10:**
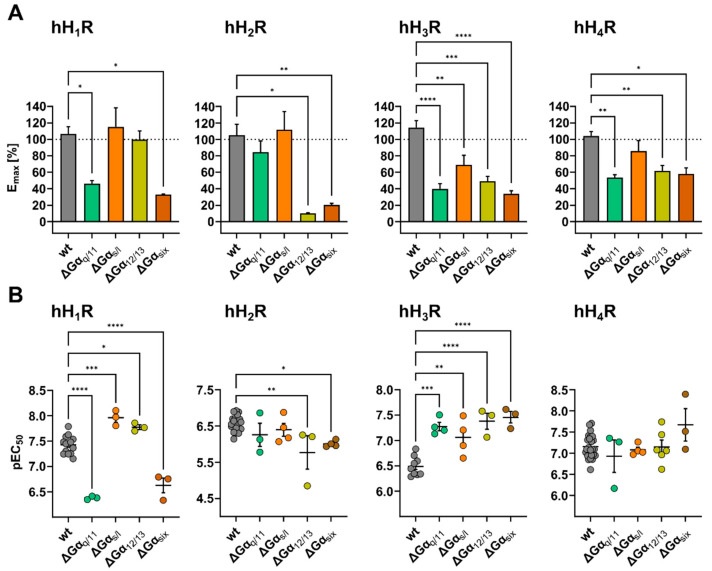
Effect of G protein knock-out on the efficacy and potency of HIS at hH_1–4_Rs. E_max_ and pEC_50_ values determined for HIS in ΔGα_x_ HEK hH_1–4_R cells. (**A**) Bar chart of E_max_ values determined for HIS in HEK hH_1–4_R cells (wt, grey) and in ΔGα_s/l_ HEK, ΔGα_q/11_ HEK, ΔGα_12/13_ HEK, ΔGα_six_ HEK cells each stably expressing hH_1–4_Rs, respectively. The E_max_ max values were calculated using AUC_60_ at the highest HIS concentration (10 µM for hH_1,2,4_R and 100 µM for hH_3_R) and normalized to the mean AUC_60_ from HEK hH_1–4_R cells at the corresponding receptor subtype (100%) and to the corresponding buffer value (0%) determined in ΔGα_x_ HEK hH_1–4_R cells. (**B**) Scatter plot of the pEC_50_ values in HEK hH_1–4_R cells (wt, grey) and in ΔGα_s/l_ HEK, ΔGα_q/11_ HEK, ΔGα_12/13_ HEK, ΔGα_six_ HEK cells each stably expressing hH_1–4_Rs, respectively. The pEC_50_ were determined by plotting the AUC_60_ against the respective HIS concentration. (**A**,**B**) Data presented are means ± SEM of at least three independent experiments each performed in triplicate. Statistical difference relative to the control was analyzed by one-way ANOVA followed by Dunnett’s multiple comparison test. Significance levels are indicated by asterisks (* *p* ≤ 0.05, ** *p* ≤ 0.01, *** *p* ≤ 0.001, **** *p* ≤ 0.0001).

A schematic illustration of the ΔGα_x_ HEK hH_1–4_R cells with regard to G protein knock-out is given in Figure 9A. The ΔGα_x_ HEK hH_1–4_R cells were stimulated with HIS at increasing concentrations and the DMR response was recorded for 60 min. Throughout, the DMR traces showed a positive deflection and were concentration dependent (Figure 9B; AUC_60_ CRCs in supplementary Figure S12). By contrast, stimulation of ΔGα_q/11_ HEK, ΔGα_12/13_ HEK and ΔGα_six_ HEK cells devoid of hH_1–4_Rs with HIS did not provoke a DMR signal. However, with ΔGα_s_ HEK cells, devoid of a HR subtype, a slight increase in the DMR signal was observed, but only at high HIS concentrations (1.00 and 10.0 µM). Therefore, we considered this DMR increase as negligible due to its low intensity (Supplementary Figure S6). To evaluate the effect of Gα protein knock-out on the DMR response, the AUC_60_ at the respective HIS concentration (hH_1,2,4_Rs 10 µM HIS and hH_3_R 100 µM HIS) using ΔGα_x_ HEK hH_1–4_R cells was compared to the mean AUC_60_ of HEK hH_1–4_R cells, in which all G proteins were present (100% control, Figure 10). This approximation was reasonable, because mostly the expression of the different receptor subtypes was comparable (Table 1).

When comparing the HIS induced DMR responses of ΔGα_s/l, 12/13_ HEK hH_1_R with that of HEK hH_1_R cells by visual inspection, there was no discernible difference (Figure 9B). Consequently, the E_max_ values for HIS using ΔGα_s/l, 12/13_ HEK hH_1_R were not significantly different from the control cell line HEK hH_1_R (Figure 10A). Of note, in Section 2.4.1 we discussed that the binding capacity of [^3^H]MEP was by factor 10 lower in ΔGα_x_ HEK hH_1_R cells than in HEK hH_1_R cells. Apparently, this difference had no impact on the signal amplitude and the E_max_ value, supporting the hypothesis that the absence of the Gα proteins impaired the binding of [^3^H]MEP to the hH_1_R [99,100]. The absence of the Gα_s_ protein in ΔGα_s/l_ HEK hH_1_R cells caused the pEC_50_ value for HIS to significantly increase to 7.96 + 0.09 compared to HEK hH_1_R cells (pEC_50_ = 7.43 + 0.05), an effect also observed in ΔGα_12/13_ HEK hH_1_R cells (pEC_50_ = 7.78 + 0.05). By contrast, the absence of the Gα_q/11_ protein in ΔGα_q/11, six_ HEK hH_1_R cells lowered the signal amplitude (E_max_ = 46 ± 38%; Figure 10A) and slightly altered the time course of the signal (Figure 9B). Moreover, the pEC_50_ value for HIS in ΔGα_q/11, six_ HEK hH_1_R cells was significantly lower in both cell lines (ΔGα_q/11_ HEK hH_1_R pEC_50_ = 6.38 ± 0.02, ΔGα_six_ HEK hH_1_R pEC_50_ = 6.63 ± 0.15) than with HEK hH_1_R cells (Figure 10B). We still hypothesize that the presence of Gα_q/11_ stabilized the active state of hH_1_R in HEK cells and that this effect is further enhanced in the absence of other Gα proteins.

The lack of Gα proteins in ΔGα_x_ HEK hH_2_R cells did not alter the time course of the DMR response (Figure 9B). Despite the lack of the Gα_s_ protein in ΔGα_s_ HEK hH_2_R cells, stimulation with HIS evoked a robust DMR response, similar to that observed with HEK hH_2_R cells. Consequently, the E_max_ value of ΔGα_s_ HEK hH_2_R cells was not significantly different compared to HEK hH_2_R cells (Figure 10A). This was unexpected, as we observed a significant decrease in E_max_ in our experiments with CTX to mask Gα_s_. Stimulation of ΔGα_q/11_ HEK hH_2_R cells with HIS showed a decrease in the E_max_ value to 84 ± 14% (Figure 10A) compared to HEK hH_2_R cells. The pEC_50_ value determined for HIS in ΔGα_s/l, q/11_ HEK hH_2_R cells remained in the same range as in HEK hH_2_R cells (Figure 10B). In Section 2.3.1 it was considered that Gα_12/13_ protein might be responsible for the HIS induced DMR at HEK hH_2_R cells. This hypothesis was affirmed as the signal amplitude of the DMR response to HIS in ΔGα_12/13_ HEK hH_2_R cells was considerably lower compared to that of HEK hH_2_R cells (Figure 9B). The corresponding E_max_ value determined in ΔGα_12/13_ HEK hH_2_R cells amounted to 10.0 ± 0.8% (Figure 10A) relative to HEK hH_2_R cells. In ΔGα_six_ HEK hH_2_R cells, which lack the Gα_12/13_ protein too, the E_max_ value was also reduced significantly to 20 ± 2.0%. In addition to E_max_, the HIS pEC_50_ value in both cell lines wassignificantly reduced (ΔGα_12/13_ HEK hH_2_R pEC_50_ = 5.77 ± 0.46; ΔGα_six_ HEK hH_2_R pEC_50_ = 6.01 ± 0.04) compared to HEK hH_2_R cells (pEC_50_ = 6.57 ± 0.05; Figure 10B). We interpreted this as an indication that Gα_12/13_ might stabilize the active state of the hH_2_R and is essential for hH_2_R mediated signal transduction in HEK cells. Further studies are necessary to substantiate or rule out the involvement of other cellular constituents, such as Gα_z_.

Unlike HEK hH_3_R cells (all G proteins present) in which the DMR signal increased steadily but slowly after addition of HIS (Figure 2A), the DMR signal in all ΔGα_x_ HEK hH_3_R cells increased rapidly, showing a peak within 10 min upon stimulation with HIS (Figure 9B). Interestingly, such a time course was not observed in any of the experiments in HEK hH_3_R cells with Gα protein modulators (Figure 5B). The E_max_ values of HIS in ΔGα_q/11_ HEK hH_3_R, ΔGα_12/13_ HEK hH_3_R cells and ΔGα_s/l_ HEK hH_3_R cells significantly declined to 40 ± 6.4%, 49 ± 5.5% and 69 ± 11%, respectively, compared to the 100% control (HEK hH_3_R cells; Figure 10A). In ΔGα_six_ HEK cells, the Gα_q/11, s/l, 12/13_ proteins were knocked-out, so it can be assumed that among the common Gα proteins, only the Gα_i/o_ was expressed. Upon stimulation of these cells with HIS, a weaker DMR response was detected with an E_max_ value of 34 ± 3.7% compared to HEK hH_3_R cells (Figure 10A). As the Gα_i/o_ signaling pathway is almost exclusively considered as physiologically relevant for the hH_3_R, we did not expect a complete suppression of the signal in ΔGα_six_ HEK hH_3_R cells. However, before assigning this response solely to the Gα_i/o_ protein, it should be noted that other G proteins, such as Gα_z_ should be considered. Strikingly, the pEC_50_ value determined for HIS in all ΔGα_x_ HEK hH_3_R cells was significantly higher (Figure 10B) compared to that determined with HEK hH_3_R cells.

Different to the hH_3_R, the time course of the HIS induced DMR response recorded using ΔGα_x_ hH_4_R cells (Figure 9) agreed well with that of HEK hH_4_R cells (100% control). The lack of Gα_q/11_ in ΔGα_q/11_ HEK hH_4_R cells led to a significant decrease in the DMR signal to 54 ± 3.3% (Figure 10A), whereas knock-out of Gα_s_ (ΔGα_s_ HEK hH_4_R) showed a weaker impact on the DMR response, reducing the E_max_ to 86 ± 13% relative to the control. This was surprising because a much more pronounced suppression was observed after treatment with CTX. In ΔGα_12/13_ HEK hH_4_R cells, the E_max_ value was suppressed to 62 ± 6.7% compared to HEK hH_4_R cells; therefore, it can be concluded that the Gα_12/13_ pathway seems to be involved in the signal transduction of the hH_4_R in HEK cells. The knock-out of the three subclasses of Gα proteins in ΔGα_six_ hH_4_R cells reduced the E_max_ value to 58 ± 7.3% compared to HEK hH_4_R cells, being in good agreement with results determined with the Gα_i/o_ modulator PTX. However, as the other three G proteins classes have been shown to be essentially involved in the signaling of the hH_4_R (see Section 2.3.1), we expected a more pronounced reduction of the signal. Perhaps the cells compensate for the lack of targeted G proteins by enhanced expression of either Gα_i/o_ or other (G) proteins are involved in the signal transduction process.

### 2.5. Pharmacological versus Molecular Biological Approach to Silence Gα Protein

The contribution of Gα proteins to the DMR response elicited by HIS at the hH_1–4_Rs stably expressed in HEK cells was investigated either by a classical pharmacological (G protein modulators) or by a molecular biological (Gα protein knock-out) approach. In the pharmacological approach, HEK hH_1–4_Rs cells were pre-treated with Gα protein modulators FR, CTX and PTX to silence either Gα_q/11_, Gα_s_ or Gα_i/o_ proteins, respectively (Section 2.3). In the molecular biological approach, the Gα_q/11_, Gα_s_, Gα_12/13_ proteins were knocked out individually or in combination (knock-out of Gα_q/11, s/l, 12/13_) using the CRISPR/Cas9 technology (Section 2.4). The focus of this section was to highlight the similarities and discuss the differences of the results obtained with these two approaches.

**Figure 11:**
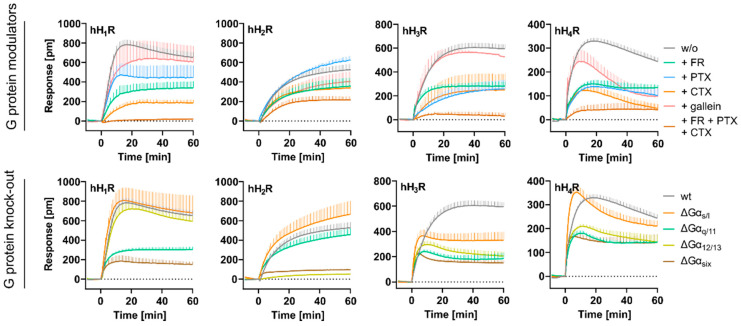
G protein inhibition using a classical pharmacological concept (G protein modulator) or by a molecular biological approach (Gα protein knock-out cells). DMR traces recorded for HIS in HEK hH_1–4_R cells in the absence (w/o modulator) or presence of G protein modulators (100 ng/mL PTX, 100 ng/mL CTX, 1 µM FR, 20 µM gallein (gal) and combination of 10 ng/mL PTX + 10 ng/mL CTX + 1 µM FR) or in Gα protein knock-out cells (ΔGα_s/l_ HEK, ΔGα_q/11_ HEK, ΔGα_12/13_ HEK and ΔGα_six_ HEK cells) stably transfected with hH_1–4_Rs. In the case of the hH_1,2,4_Rs the cells were stimulated with 10 µM HIS, whereas in the case of the hH_3_R the cells were stimulated with 100 µM HIS. All traces shown were corrected for the assay buffer and represent means ± SEM of at least three independent experiments, each performed in triplicate.

Silencing of the Gα_q/11_ signaling pathway either by 1.00 µM FR in HEK hH_1–4_R cells or by knocking out Gα_q/11_ in ΔGα_q/11_ HEK hH_1–4_R cells have shown agreement in terms of E_max_ and pEC_50_ values for HIS. At the hH_1_R, for example, the time course of DMR traces were similarly altered by both approaches compared to the control (HEK hH_1_R cell w/o; Figure 11) and the E_max_ for HIS was significantly reduced (ΔGα_q/11_ HEK hH_1_R E_max_ = 46 ± 3.8; HEK hH_1_R + 1 µM FR E_max_ = 41 ± 9.5) relative to HEK hH_1_R cells (100% w/o modulator). Moreover, both procedures to silence the Gα_q/11_ have led to a significant decrease in the pEC_50_ determined for HIS (ΔGα_q/11_ HEK hH_1_R pEC_50_ = 6.38 ± 0.02; HEK hH_1_R + 1 µM FR pEC_50_ = 6.60 ± 0.29) relative to HEK hH_1_R control cells (pEC_50_ = 7.43 ± 0.05). A similar effect was observed for HIS with ΔGα_six_ HEK hH_1_R cells, which also lack the Gα_q/11_ protein. We expected such a pronounced perturbation of the E_max_ and the pEC_50_ value, as hH_1_R is predominantly described as a Gα_q/11_ coupled receptor [57]. It was surprising that for the hH_2_R, silencing of the Gα_q/11_ signaling pathway by both approaches (FR and Gα_q/11_ knock-out) had almost no impact on the DMR response (kinetics, E_max_ and pEC_50_), as it is commonly accepted that the Gα_q/11_ protein is considerably involved in the signal transduction of the hH_2_R [56]. We cannot confirm this in the DMR assay using HEK cells. By contrast, deactivation of the Gα_q/11_ signaling pathway either by FR or knock-out affected the E_max_ value for HIS at hH_3,4_Rs (Figure 11). In HEK hH_3,4_Rs, 1.00 µM FR reduced the E_max_ to 57 ± 5.4% and 47 ± 4.5%, respectively (Figure 6A) and in ΔGα_q/11_ HEK hH_3,4_R cells the E_max_ was diminished to 40 ± 6.4% and 54 ± 3.3%, respectively (Figure 10A; compared to untreated HEK hH_3,4_R cells). Moreover, at hH_3_R, the pEC_50_ value for HIS increased significantly by both approaches (HEK hH_3_R + 1 µM FR pEC_50_ = 7.20 ± 0.05, ΔGα_q/11_ HEK hH_3_R cells pEC_50_ = 7.28 ± 0.08) relative to HEK hH_3_R cells (pEC_50_ = 6.49 ± 0.06). By contrast, in both systems the pEC_50_ determined for HIS at the hH_4_R was not significantly altered. As both approaches led to the same consequences, we are convinced that the results are not an artifact and conclude that Gα_q/11_ contributed to the DMR signaling of the hH_3,4_Rs in HEK cells, even though the limited literature suggests the opposite [91,101]. We consider this as an intriguing starting point for further investigations.

Unlike Gα_q/11_, we found differences between CTX and knocking-out Gα_s_ (Figure 11). To be more specific, in experiments using HEK hH_1–4_Rs cells pre-treated with 100 ng/mL CTX, we concluded that Gα_s_ was markedly involved in the hH_1–4_R mediated signal transduction process in HEK cells throughout. For example, for the hH_1_R the E_max_ was dramatically reduced to 23 ± 4.9% (Figure 6A). Moreover, the E_max_ of HIS determined in HEK hH_3,4_R cells was significantly reduced to 54 ± 7.6% or 35 ± 7.9%, respectively (Figure 5 and Figure 6A). By contrast, we observed that knock-out of Gα_s/l_ in ΔGα_s/l_ HEK hH_1–4_R cells had a weaker effect on the E_max_ value. In ΔGα_s/l_ HEK hH_1,3,4_R cells, the E_max_ value amounted to 115 ± 23%, 69 ± 12% and 86 ± 13% of control responses respectively (Figure 10A), suggesting that Gα_s_ plays a supporting role in the HIS induced DMR response. Various explanations can be considered to address this discrepancy in E_max_ between the two approaches. On the one hand, it seems possible that HEK cells have “adapted” their repertoire of expressed Gα proteins to compensate for the lack of Gα_s_ in ΔGα_s/l_ HEK hH_1–4_R cells so that the E_max_ remained unaffected. On the other hand, it is conceivable that, in addition to Gα_s_, CTX may have off-target effects that were relevant for the generation of the DMR signal in HEK hH_1–4_R cells, which led to a decrease in E_max_. Elucidation of the difference in results between the pharmacological and molecular biological approaches for Gα_s_ modulation should be pursued in the future.

Regarding the Gα_i/o_ signaling pathway, in the experiments with PTX we found that Gα_i/o_ was directly involved in the hH_1_R mediated DMR response in HEK cells, as the E_max_ was reduced to 50 ± 9.3% in the presence of 100 ng/mL PTX (Figure 6A). Alternatively, in ΔGα_six_ hH_1_R cells, which among the canonical Gα proteins only express Gα_i/o_, HIS elicited a DMR response with a corresponding E_max_ of 33 ± 0.5% (Figure 10A). As we ruled out that Gα_12/13_ and Gα_z_ play a role in the hH_1_R mediated DMR response (Figure 7), we conclude that the residual 33% represent the interaction of the hH_1_R with the Gα_i/o_, in accordance with literature [57]. The hH_3,4_Rs are considered as Gα_i/o_ selective receptors [57], however, according to our experiments, we conclude that Gα_i/o_ was not exclusively involved in the manifestation of the DMR signal in HEK hH_3,4_R cells. Namely, pretreatment of HEK hH_3,4_R cells with 100 ng/mL of PTX led to a dramatic decrease in E_max_, and the pEC_50_ was reduced in HEK hH_3_R cells compared to controls (Figure 6B). Additionally, in the presence of the Gα_q/11_ protein modulator FR, the E_max_ was significantly reduced for both receptor subtypes (Figure 6B). Moreover, it was not possible to completely abolish the HIS triggered DMR in HEK hH_3,4_R cells with a modulator cocktail comprising FR, CTX and PTX (Figure 7). In addition to the canonical Gα proteins, we observed that the Gα_12/13_ proteins might be involved in the signal transduction process of the hH_3,4_Rs, as the E_max_ in ΔGα_12/13_ HEK hH_3,4_R cells decreased by about 55% compared with HEK hH_3,4_R cells (Figure 10A). However, we cannot exclude that the Gα_z_ might also be involved. In the case of the hH_2_R, the modulation of Gα_i/o_ by 100 ng/mL PTX had a weaker effect on the HIS induced DMR response (E_max_ = 77 ± 4.1%) compared to the hH_1,3,4_Rs (E_max_ 51–32%; Figure 6A). Moreover, we failed to suppress the HIS induced DMR response by more than 40% with Gα protein modulators FR, CTX and PTX (Figure 7), and most of the DMR signal was abolished in ΔGα_12/13_ HEK hH_2_R and ΔGα_six_ HEK hH_2_R cells, both of which lack the Gα_12/13_ protein (Figure 10A). We conclude that Gα_q/11_, Gα_s_, and Gα_i/o_ played a minor role in the generation of the HIS DMR signal in HEK hH_2_R cells, and that Gα_12/13_ proteins must have been involved. It has already been described in the literature that the hH_2_R is capable to interact with the Gα_12/13_ protein [59,60], however, it was unexpected that the involvement of Gα_12/13_ would exceed the contribution of Gα_q/11_, Gα_s_, and Gα_i/o_.

In summary, we successfully established a DMR assay for the entire histaminergic receptor family stably expressed in HEK cells, providing an opportunity to monitor the functions of HRs and its ligands in real-time. High S/B-ratios above 100 for HIS and 24 for inverse agonists facilitate investigations on signaling pathways of hH_1–4_Rs and might be beneficial for further investigations, e.g., with respect to inverse agonism and functional bias of HR ligands. We took advantage of the integrative nature of the DMR assay to investigate the involvement of endogenously expressed G proteins in the signaling transduction processes mediated by hH_1–4_Rs. However, in view of the physiological relevance of the results, experiments with cells or tissues which endogenously express the receptors are pending. For example, using modulatory tools such as PTX, CTX and FR, the impact of ligands on the signaling pathway of the receptor can be studied as well, which is particularly interesting with respect to ligand induced signal bias. At this point, it should be noted that apart from G proteins, the recruitment of β-arrestin also plays an important role in the signal transduction processes of GPCRs [24] and consequently also for HRs [102,103,104]. Interestingly, although investigations on the mechanistic details of β-arrestin activation are available, there is also evidence that no β-arrestin mediated signaling was observed in absence of functional G proteins [41]. The DMR assay could be a valuable approach to investigate the contribution of β-arrestins to a holistic response of HRs. Pharmacological tools (e.g., biased ligands, protein inhibitors) in combination with a molecular biological approach (e.g., cells lacking (either) Gα proteins and/or β-arrestins) might be helpful to gain new insights into the interaction of G proteins and β-arrestins [41]. Moreover, several polymorphisms were discovered for HRs [105] which are under investigation to be associated with diseases such as heart failure (H_2_R [106]) or allergic rhinitis (H_4_R [107]) and correlated with the effectiveness of drugs (H_1_R [108], H_3_R [109], H_4_R [110]). Thus, the DMR assay might be a valuable tool to characterize such polymorphisms of HRs, especially focusing on the differences in the signaling pathways between receptor variants.

Although our studies still leave some open questions, we are convinced that the presented work provides valuable information for further investigation on signal transduction mechanisms of the HR family.

## 3. Materials and Methods

### 3.1. Materials

Dulbecco’s modified Eagle’s medium with phenol red (DMEM), L-glutamine solution (200 mM) and penicillin-streptomycin solution (10,000 units penicillin and 10 mg streptomycin per mL in 0.9% NaCl) were purchased from Sigma-Aldrich (Taufkirchen, Germany). Hanks’ Balanced Salt Solution (HBSS) and Leibovitz’ L-15 medium (L-15) were from Fisher Scientific (Nidderau, Germany). FBS, and geneticin (G418) were from Merck Biochrom (Darmstadt, Germany). Trypsin/EDTA was either from Merck Biochrom (Darmstadt, Germany) or from VWR International GmbH (Ismaning, Germany). The pIRESpuro3 vector was a gift from Prof. Dr. Gunter Meister (Biochemistry I, University of Regensburg, Regensburg, Germany). Histamine dihydrochloride (HIS), was from Fisher Scientific (Schwerte, Germany). Diphenhydramine hydrochloride (DPH), mepyramine maleate (MEP) and famotidine (FAM) were from Sigma (Taufkirchen, Germany). Thioperamide maleate (THIO), UR-DE257 (DE257) and JNJ7777120 (JNJ) were synthesized in-house according to standard procedures. Pitolisant hydrochloride (PIT) was kindly provided by Prof. Dr. Katarzyna Kiec-Kononowicz (Jagiellonian University, Krakow, Poland). The ligands were dissolved in Millipore water, except for famotidine (FAM), which was dissolved in DMSO (Merck, Darmstadt, Germany). FR900359 (UBO-QIC) was purchased from the Institute of Pharmaceutical Biology, University of Bonn (Bonn, Germany). Pertussis Toxin (PTX) was purchased from Bio-Techne GmbH (Wiesbaden, Germany) and Cholera Toxin from Enzo Life Sciences (Lörrach, Germany). Gallein was from Santa Cruz Biotechnology (Heidelberg, Germany).

### 3.2. Cell Culture

#### 3.2.1. Parental Cells and General Culture Conditions

HEK293T cells were a kindly provided by Prof. Dr. Wulf Schneider (Institute for Medical Microbiology and Hygiene, Regensburg, Germany). HEK293T and CRISPR/Cas9 edited HEK293A lacking the Gα proteins Gα_s_ + Gα_olf_ (ΔGα_s/l_ HEK) [63], Gα_q_ + Gα_11_ (ΔGα_q/11_ HEK) [44], Gα_12_ + Gα_13_ (ΔGα_12/13_ HEK) [64] or Gα_q_ + Gα_olf_ + Gα_11_ + Gα_s_ + Gα_12_ + Gα_13_ (ΔGα_six_ HEK) [41] were maintained in DMEM supplemented with 10% FBS, penicillin (100 U/mL) and streptomycin (100 µg/mL) (P/S) at 37 °C in a water-saturated atmosphere containing 5% CO_2_. Cells were periodically monitored for mycoplasma contamination by means of the Venor GeM Mycoplasma Detection Kit (Minerva Biolabs, Berlin, Germany) and proven negative.

#### 3.2.2. Generation of HEK hH_1–4_R Cells

The HEK hH_1_R, HEK hH_2_R, HEK hH_3_R and HEK hH_4_R cells are abbreviated designations of the previously described stable single clone transfectants: HEK293T-SP-FLAG-hH_1_R K12 [111], HEK293T-SP-FLAG-hH_2_R K46 [111], HEK293T-SP-FLAG-hH_3_R K16 [89] and HEK293T-SP-FLAG-hH_4_R K3 cells [89], respectively. The procedures for molecular cloning of the receptors and the generation of the stable cell lines are described elsewhere [89,111]. The cells were cultured in DMEM supplemented with 10% FBS + P/S and 600 µg/mL G418.

#### 3.2.3. Generation of ΔGα_x_ HEK hH_1–4_R Cells

For the generation of ΔGα_x_ HEK hH_1–4_R cells CRISPR/Cas9 modified HEK293A cells lacking the Gα proteins Gα_s_ + Gα_olf_ (ΔGα_s/l_ HEK) [63], Gα_q_ + Gα_11_ (ΔGα_q/11_ HEK) [44], Gα_12_ + Gα_13_ (ΔGα_12/13_ HEK) [64] or Gα_q_ + Gα_olf_ + Gα_11_ + Gα_s_ + Gα_12_ + Gα_13_ (ΔGα_six_ HEK) [41] were transfected with the pIRESneo3-SP-FLAG vector encoding the hH_1–4_Rs according to the procedure described for HEK hH_4_R cells [89] except that no single clone selection was performed. The cells were cultured in DMEM supplemented with 10% FBS + P/S and 600 µg/mL G418.

### 3.3. Methods

#### 3.3.1. Radioligand Binding

All radioligand binding experiments (saturation and competition) were performed using suspensions of live HEK hH_1–4_R and ΔGα_x_ HEK hH_1–4_R cells. The cells were cultivated in DMEM supplemented with 10% FBS + P/S and 600 µg/mL G418 until 90–100% confluency was reached. On the day of the assay, the cells were detached by trypsinization (0.05% trypsin, 0.02% EDTA in PBS, at 37 °C for 2–4 min), harvested by centrifugation (800× *g* at rt for 5 min) and resuspended in L-15 medium devoid of additional supplements. The number of cells was determined using a hemocytometer (Neubauer, improved) and the cell density was adjusted to 1.0 × 10^6^ cells/mL.

Before dispensing the cell suspension, all (radio)ligand dilutions were prepared 10-fold concentrated in L-15 medium and dispensed (10 µL/well) in 96 well plates (PP microplates, Greiner Bio-One, Frickenhausen, Germany). Total binding was determined in the presence of L-15 medium (10 µL/well), and the non-specific binding was assessed in the presence of a competitor: for hH_1_R diphenhydramine (DPH), for hH_2_R famotidine (FAM), for hH_3,4_Rs histamine (HIS), each at a final concentration of 10 µM. For saturation binding experiments, serial dilutions of the following radioligands were prepared (10 µL/well): [^3^H]MEP (a_s_ = 20 Ci/mM, Hartmann Analytics GmbH, Braunschweig, Germany) for the hH_1_R, [^3^H]UR-DE257 (a_s_ = 32.9 Ci/mmol) [69] for the hH_2_R and [^3^H]UR-PI294 (a_s_ = 93.3 Ci/mmol) [70] for the hH_3,4_Rs. For competition binding experiments, dilutions of “cold” ligands (10 µL/well) were incubated in the presence of 5 nM [^3^H]MEP for the hH_1_R, 50 nM [^3^H]UR-DE257 for the hH_2_R, 2 nM [^3^H]UR-PI294 for the hH_3_R and 5 nM [^3^H]UR-PI294 for the hH_4_R.

Subsequently, the cell suspension was added to the (radio)ligands (80 µL/well) to reach a final assay volume of 100 µL/well. After an incubation period of 60–120 min, the cells were harvested by filtration using a Brandel 96 sample harvester and the radioactivity was determined by liquid scintillation counting as described previously [112].

Data was analyzed using the GraphPad Prism 8 or 9 software (San Diego, CA, USA). Specific binding was calculated by subtracting the non-specific binding from the total binding. For saturation binding experiments binding data was plotted against the free radioligand concentration (nM) and best fitted to a one site saturation binding model (one site—total and non-specific binding; one site—specific binding) yielding K_d_ values. Receptor expression was quantified using the extrapolated B_max_ values, specific activity (a_s_) of the radioligands and the cell number seeded per well and is indicated as specific binding sites per cell.

For competition binding experiments, the specific binding was plotted against the −log(concentration ligand) and analyzed applying the four parameters logistic equation (log(modulator) vs. response—variable slope (four parameters)) yielding the p*IC*_50_ values, which were individually converted to pKi values using the Cheng–Prusoff equation [113].

#### 3.3.2. DMR Assay

The DMR assay was essentially performed as described [38] with the following modifications: The cells were cultured in DMEM supplemented with 10% FBS, 2 mM L-glutamine, P/S, and 600 µg/mL G418 until 90–100% confluency. The day before the assay, the cells were detached by trypsinization (0.05% trypsin, 0.02% EDTA in PBS, at 37 °C for 2–4 min), harvested by centrifugation (800× *g*, RT, 5 min,) and subsequently resuspended in DMEM supplemented with 10% FBS + P/S w/o G418. The cell density was adjusted to 1 × 10^6^ cells/mL and the cell suspension was dispensed (90 µL/well) into an uncoated label-free 96 well plate (Cat. No. 5080, Corning B.V. Life Sciences, Amsterdam, Netherlands). Subsequently, the cells were spun down at 600× *g* for 1 min and allowed to attach in a humidified atmosphere containing 5% CO_2_ at 37 °C overnight. On the day of the measurement, the cells were gently washed twice with assay medium (HBSS containing 20 mM HEPES). After the last washing step, the final volume was adjusted to 90 µL/well with assay medium and the plate was centrifuged at 600× *g* for 1 min. The cells were allowed to equilibrate at 37 °C for at least 2 h in an EnSpire multimode plate reader (PerkinElmer, Rodgau, Germany), before the baseline was recorded every minute for 5–10 min. Immediately after the baseline record, the compounds (10 µL/well; 10-fold concentrated in assay medium) were added and the response was recorded every minute for 60 min.

For experiments with the G-protein modulators PTX and CTX the cells were pretreated with the modulator at the respective final concentration (1.00, 10.0 or 100 ng/mL) overnight and the assay was performed as described above. In the case of FR900359 and gallein the cells were incubated with the modulator (FR900359 1.00, 0.10 or 0.01 µM; gallein 20.0 µM) for 30 min before the baseline record. Afterwards the assay was performed by analogy with the procedure described above.

The time course data is presented as resonance wave-length shift in pm relative to the last data point before the test compounds were added at time zero. Data were analyzed using the GraphPad Prism 8 and 9 software (San Diego, CA, USA). For analysis, the data were corrected for the baseline drift by subtracting the mean values of the buffer control. Subsequently, the area under curve (AUC) was calculated individually for each well defining the first 5–10 values as baseline. For the estimation of the S/B ratios the modulus of the AUC was used according to the following equation.

$$\frac{S}{B}\mathrm{ratio}\;=\;\frac{\left|{\mathrm{AUC}}_{\mathrm{signal}}\right|}{\left|{\mathrm{AUC}}_{\mathrm{buffer}}\right|}$$

Corresponding to the signal deflection (positive or negative) the positive or the negative AUC was used for the construction of concentration response curves. The AUCs were normalized to the maximum response elicited by the highest histamine concentration (100% control) and assay medium (0% control) and plotted against the logarithmic ligand concentration. The pEC_50_ values were calculated by applying the four parameters logistic equation (log(agonist) vs. response—variable slope (four parameters)). Real-time DMR traces are presented from representative experiments (mean ± SEM) with each trace reflecting the average of three technical replicates. Each experiment was performed at least three times to obtain at least three independent biological replicates.

### 3.4. Statistical Analyses

Statistical differences were analyzed using either the student *t* test (two-tailed) or one-way ANOVA followed by Dunnett’s or Tukey’s multiple comparisons test, as indicated in the corresponding Figures/Tables. All calculated *p*-values are two-sided and considered as statistically significant when lower than 0.5 indicated as * *p* ≤ 0.05, ** *p* ≤ 0.01, *** *p* ≤ 0.001, **** *p* ≤ 0.0001. All calculations were performed using the GraphPad Prism 8 or 9 software (San Diego, CA, USA).

## Figures and Tables

**Table 1:** Radioligand saturation binding data determined with live HEK hH_1–4_R and ΔGα_x_ HEK hH_1–4_R cells using [^3^H]MEP, [^3^H]UR-DE257 or [^3^H]UR-PI294 as radiolabeled tracers for hH_1_R, hH_2_R or hH_3,4_R, respectively.

G Protein Knock-Out (Δ)	HR	Binding Sites/Cell	pK_d_	*n*	pK_d_ Ref.
none	hH_1_R	2.50 × 10^6^ + 0.52 × 10^6^	8.32 + 0.08	4	8.36 ^a^
ΔGα_s/l_	hH_1_R	6.16 × 10^5^ + 2.07 × 10^5^	8.42 + 0.04	3	
ΔGα_q/11_	hH_1_R	1.78 × 10^5^ + 0.26 × 10^5^	8.61 + 0.18	3	
ΔGα_12/13_	hH_1_R	2.18 × 10^5^ + 0.04 × 10^5^	8.46 + 0.08	3	
ΔGα_six_	hH_1_R	Not detectable		3	
none	hH_2_R	2.43 × 10^6^ + 0.23 × 10^6^	7.19 + 0.06	6	7.26 ^b^
ΔGα_s/l_	hH_2_R	1.68 × 10^6^ + 0.33 × 10^6^	7.37 + 0.19	3	
ΔGα_q/11_	hH_2_R	9.37 × 10^6^ + 0.63 × 10^6^	7.40 + 0.06	3	
ΔGα_12/13_	hH_2_R	3.94 × 10^6^ + 0.12 × 10^6^	7.98 + 0.05 ****	3	
ΔGα_six_	hH_2_R	4.23 × 10^6^ + 0.03 × 10^6^	7.86 + 0.06 ***	3	
none	hH_3_R	1.01 × 10^5^ + 0.21 × 10^5^	8.61 + 0.03	3	8.96 ^c^
ΔGα_s/l_	hH_3_R	3.20 × 10^4^ + 0.83 × 10^4^	8.71 + 0.11	3	
ΔGα_q/11_	hH_3_R	3.94 × 10^4^ + 0.93 × 10^4^	8.73 + 0.02	2	
ΔGα_12/13_	hH_3_R	4.63 × 10^4^ + 1.32 × 10^4^	8.49 + 0.10	3	
ΔGα_six_	hH_3_R	1.01 × 10^5^ + 0.32 × 10^5^	8.41 + 0.18	3	
none	hH_4_R	1.37 × 10^5^ + 0.18 × 10^5^	8.45 + 0.05	4	8.26 ^d^
ΔGα_s/l_	hH_4_R	1.42 × 10^5^ + 0.33 × 10^5^	8.26 + 0.07	4	
ΔGα_q/11_	hH_4_R	1.24 × 10^5^ + 0.11 × 10^5^	8.54 + 0.02	3	
ΔGα_12/13_	hH_4_R	5.23 × 10^4^ + 0.42 × 10^4^	8.55 + 0.06	3	
ΔGα_six_	hH_4_R	1.06 × 10^5^ + 0.20 × 10^5^	8.22 + 0.08	4	

Data is presented as means ± SEM of at least three independent experiments, each performed in triplicate. Reference data was transformed from K_d_ to pK_d_ values. ^a^ Saturation binding experiments with live HEK-CRE-Luc hH_1_R hMSR1 cells and [^3^H]MEP [20]. ^b^ Saturation binding experiments with live HEK-CRE-Luc hH_2_R cells and [^3^H]UR-DE257 [69]. ^c^ Saturation binding experiments with membrane preparations of Sf9 insect cells co-expressing the hH_3_R + Gα_i_ + Gβγ and [^3^H]UR-PI294 [70]. ^d^ Saturation binding experiments with membrane preparations of Sf9 insect cells co-expressing the hH_4_R + Gα_i_ + Gβγ and [^3^H]UR-PI294 [70]. Statistical difference in pK_d_ value among HR subtypes relative to the control (e.g., HEK hH_1_R (control) versus ΔGα_x_ HEK hH_1_R) was analyzed by one-way ANOVA followed by Dunnett’s multiple comparison test calculated as *** *p* ≤ 0.001, **** *p* ≤ 0.0001.

**Table 2:** Summary of binding and functional data determined on live HEK hH_1–4_R cells and reference data.

		Determined			References			
		Comp Bdg	DMR		miniG Recruitment [80]		Luciferase Reporter Gene	
Rcptr.	Cpd.	pK_i_	pEC_50_	E_max_	pEC_50_/(pK_b_)	E_max_	pEC_50_/(pK_b_)	E_max_
hH_1_R	HIS	3.37 ± 0.29	7.38 ± 0.05	100	6.16 ± 0.09	100	6.87 ± 0.06 [20]	100 [20]
	DPH	7.80 ± 0.17	7.82 ± 0.07	−22 ± 3	6.95 ± 0.04	−4 ± 0.1	(7.66 ± 0.24) [20]	-
hH_2_R	HIS	4.32 ± 0.38	6.57 ± 0.05	100	6.94 ± 0.05	100	6.49 ± 0.27	100
	FAM	7.75 ± 0.33	7.37 ± 0.06	−10 ± 5	7.29 ± 0.10	−9 ± 0.7	7.47 ± 0.15	n.d.
hH_3_R	HIS	6.80 ± 0.19	6.49 ± 0.06	100	6.47 ± 0.04	100	8.48 ± 0.09 [89]	100 [89]
	PIT	8.72 ± 0.05	7.02 ± 0.22	−21 ± 2	(8.41 ± 0.05)	-	n. d.	n. d.
hH_4_R	HIS	7.25 ± 0.05	7.15 ± 0.05	100	6.40 ± 0.04	100	7.77 ± 0.12 [23]	100 [23]
	THIO	6.66 ± 0.12	7.04 ± 0.14	−45 ± 6	6.68 ± 0.04	−8 ± 1.9	6.92 ± 0.10 [23]	−32.0 ± 0.04 [23]

**Competition binding (Comp. Bdg):** The pKi values for histamine (HIS), diphenhydramine (DPH), famotidine (FAM), pitolisant (PIT) and thioperamide (THIO) were determined with live HEK hH_1–4_R cells in the presence of 5 nM [^3^H]mepyramine ([^3^H]MEP) at the hH_1_R, 50 nM [^3^H]UR-DE257 at the hH_2_R, 2 nM or 5 nM [^3^H]UR-PI294 at the hH_3_R or hH_4_R, respectively. **DMR:** The pEC_50_ and E_max_ values were determined by converting the DMR traces to CRCs using the positive AUC_60_ for HIS or the negative AUC_60_ for the inverse agonists (DPH at the hH_1_R, FAM at the hH_2_R, PIT at the hH_3_R and THIO at the hH_4_R). These values were subsequently normalized to AUC_60_ for the buffer (0%) and the respective highest histamine concentration (100%). The negative sign of E_max_ values for inverse agonists implies a negative deflection of the originate DMR traces (Figure 2C). All values represent means ± SEM of at least three independent experiments, each performed in triplicate. n. d. means not determined.

## Data Availability

The data presented in this study are available on request from the first author.

## Supplementary Materials

The following supplementary Materials are available online at https://www.mdpi.com/article/10.3390/ijms22189739/s1.
