# Supplementary material for: Label-Free Investigations on the G Protein Dependent Signaling Pathways of Histamine Receptors
Source: Int J Mol Sci. 2021 Sep 9;22(18):9739. doi: 10.3390/ijms22189739 (PMC8467282; doi:10.3390/ijms22189739)
Supplement: Supplementary file 1 [file ijms-22-09739-s001.zip › SM_Seibel-Ehlert et al_DMR for hH1-4Rs_IJMS.pdf]

Supplementary Materials

Label-free investigations on the G protein dependent signaling pathways of histamine receptors

Ulla Seibel-Ehlert [1*], Nicole Plank [1], Asuka Inoue [2], Guenther Bernhardt [1] and Andrea Strasser [1,*]

[1] Institute of Pharmacy, Faculty of Chemistry and Pharmacy, University of Regensburg, 93040 Regensburg, Germany; ulla.seibel@ur.de (U.S.-E.); nicole.plank@ur.de (N.P.); guenther.bernhardt@ur.de (G.B.); andrea.strasser@ur.de (A.S.)

[2] Department of Pharmacological Sciences, Tohoku University, Sendai, 980-8578, Japan; iaska@tohoku.ac.jp

* Correspondence: ulla.seibel@ur.de (U.S.-E.); andrea.strasser@ur.de (A.S.) Tel.: +49-941-943-2960 (U.S.-E.); +49-941-943-4821 (A.S.)

Contents

Figure S1	Structures of HR ligands investigated in the DMR assay	p. 3
Figure S2	Representative saturation binding curves determined on live HEK $hH_{1-4}R$ cells	p. 4
Figure S3	Representative saturation binding curves determined on live $\Delta G\alpha_x$ HEK $hH_{1-4}R$ cells	p. 5
Figure S4	Radioligand displacement curves determined for histamine (HIS) (blue) and a receptor specific inverse agonist (red) using live HEK $hH_{1-4}R$ cells	p. 6
Table S1	Comparison of results from radioligand displacement experiments performed on live HEK $hH_{1-4}R$ cells with reference data	p. 7
Table S2	Radioligand competition binding experiments	p. 8
Figure S5	Magnified illustration of the signal dip observed in HEK hH_2R cells upon stimulation with HIS, which was not observed in HEK $hH_{1,3,4}R$ cells.	p. 9
Figure S6	Lack of ligand induced DMR response in non-transfected HEK293T wildtype (wt) cells	p. 10
Figure S7	Constitutive activity of $hH_{1-4}Rs$ stably expressed in HEK cells.	p. 11
Text S1	Impact of the time interval used for calculations of AUC on S/B ratios	p. 12
Figure S8	Impact of the time interval used for AUC calculation on the signal-to-background (S/B) ratio for histamine (red) and receptor specific inverse agonists (blue) at $hH_{1-4}Rs$.	p. 12
Table S3	Impact of the time intervals used for AUC calculations on the S/B values determined for histamine and inverse agonists in the DMR assay using HEK $hH_{1-4}R$ cells	p. 13
Figure S9	Impact of the time interval used for AUC calculation on the CRCs for histamine and inverse agonists at $hH_{1-4}Rs$.	p. 14
Table S4	Impact of the time interval used for the calculations of AUC on the pEC_{50} and E_{max} values for histamine and the corresponding inverse agonist at $hH_{1-4}Rs$.	p. 15
Figure S10	CRCs determined for histamine (HIS) using HEK $hH_{1-4}R$ cells in the absence and the presence of G protein inhibitors	p. 16
Figure S11	E_{max} and pEC_{50} values determined for HIS in the absence and the presence of G protein inhibitors	p. 17
Figure S12	CRCs determined for histamine (HIS) using $\Delta G\alpha_x$ HEK $hH_{1-4}R$ cells	p. 18
	References	p. 19

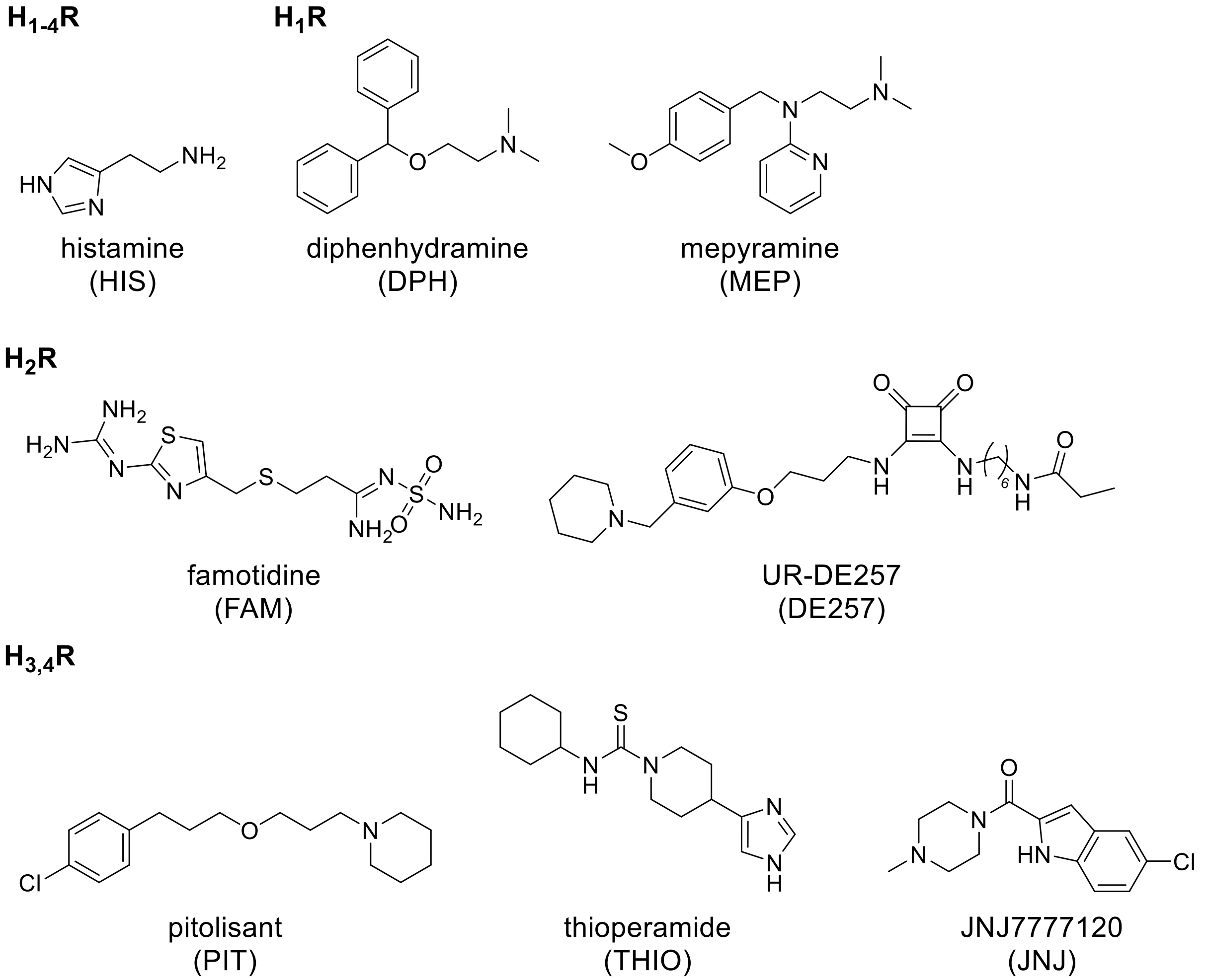

Figure S1. Structures of HR ligands investigated in the DMR assay

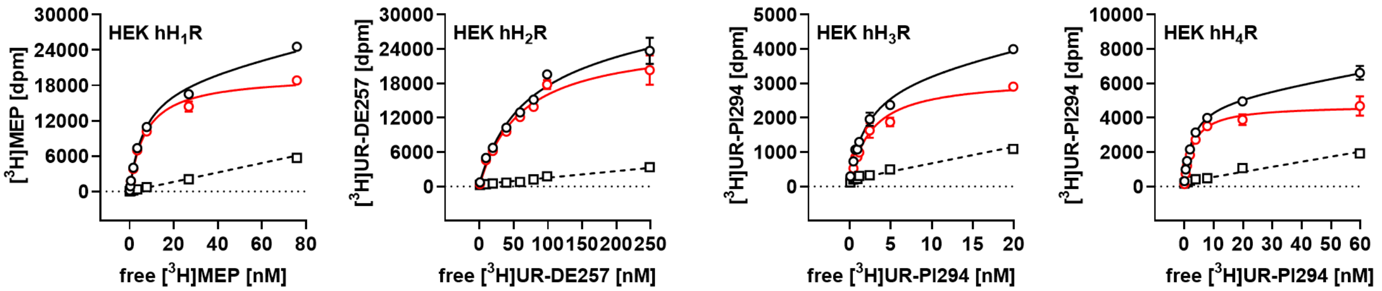

Figure S2. Representative saturation binding curves determined on live HEK $hH_{1\text{-}4}R$ cells. For the experiments using live cells the following radiolabeled tracers were used: $[^3H]$mepyramine ($[^3H]$MEP) for HEK hH_1R, $[^3H]$UR-DE257 for HEK hH_2R and $[^3H]$UR-PI294 for HEK $hH_{3,4}R$. The non-specific binding was determined in the the presence of diphenhydramine (HEK hH_1R), famotidine (HEK hH_2R) or histamine (HEK $hH_{3,4}Rs$), each at a final concentration of 10 μM. Each point represents mean ± sem of a technical triplicate from a representative experiment. Error bars of specific binding were calculated according to the Gaussian low of error propagation.

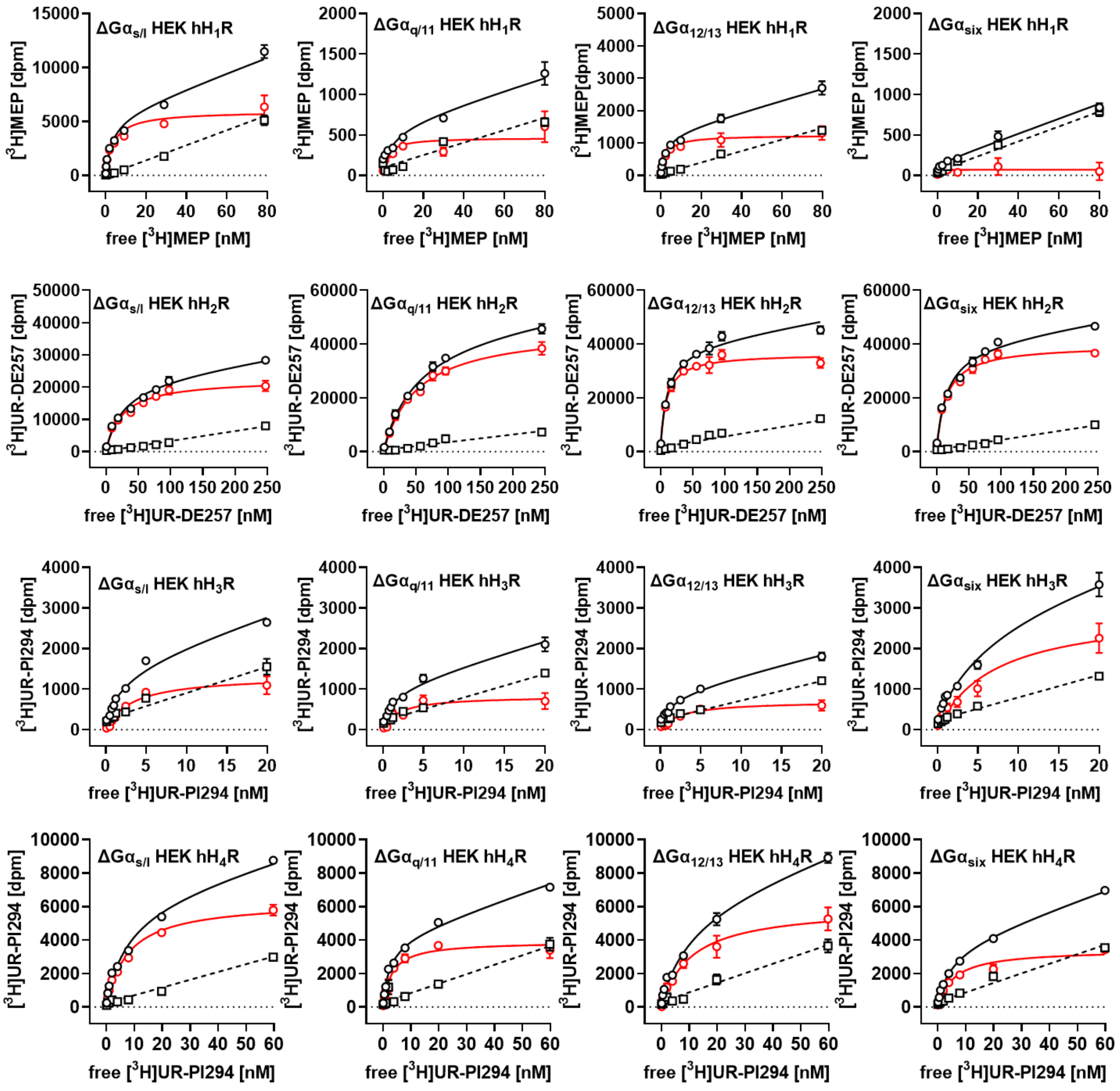

Figure S3. Representative saturation binding curves determined on live $\Delta G\alpha_x$ HEK hH$_{1\text{-}4}$R cells. For the experiments using live cells, the following radiolabeled tracers were used: [^{3}H]mepyramine ([^{3}H]MEP) for hH$_1$R, [^{3}H]UR-DE257 for hH$_2$R and [^{3}H]UR-PI294 for hH$_{3,4}$R. The non-specific binding was determined in the presence of diphenhydramine (HEK hH$_1$R), famotidine (HEK hH$_2$R) or histamine (HEK hH$_{3,4}$Rs), each at the final concentration of 10 μM. Each point represents mean ± sem of the technical triplicate from a representative experiment. Error bars of specific binding were calculated according to the Gaussian low of error propagation.

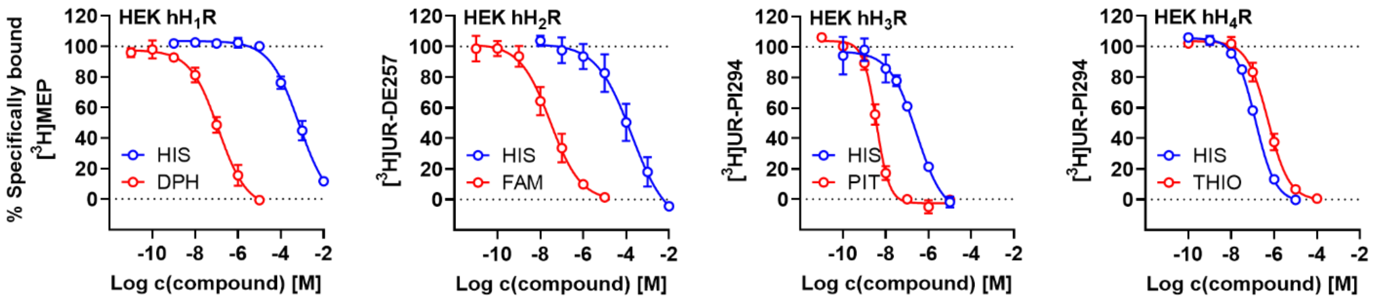

Figure S4. Radioligand displacement curves determined for histamine (HIS) (blue) and a receptor specific inverse agonist (red) using live HEK $hH_{1-4}R$ cells. Cold ligands (HIS, diphenhydramine (DPH), famotidine (FAM), pitolisant (PIT) and thioperamide (THIO)) were incubated at indicated concentrations in presence of 5 nM $[^3H]$mepyramine ($[^3H]$MEP) on HEK hH_1R, 50 nM $[^3H]$UR-DE257 on HEK hH_2R, 2 nM or 5 nM $[^3H]$UR-PI294 on HEK hH_3R or hH_4R, respectively. The non-specific binding was determined in the presence of DPH (hH_1R), FAM (hH_2R) or HIS ($hH_{3,4}Rs$), each at the final concentration of 10 μM. The non-specific binding was subtracted from the total binding to obtain the specific binding. Specific binding was normalized to the buffer value (100%) and the corrected non-specific binding value (0%). Each point represents mean ± sem of at least three independent experiments, each performed in triplicate.

Table S1. Comparison of results from radioligand competition binding experiments performed on live HEK $hH_{1-4}R$ cells with reference data.

		pK_i determined		Reference pK_i		
		Whole cells [³H]	n	Whole cells [³H]	Whole cells [BRET]	Cell membranes/ homogenates [³H]
HEK hH_1R	HIS	3.37 ± 0.29	4			4.2[1], 5.62[2], 5.9[3]
	DPH	7.80 ± 0.17	3	7.40[4]		7.9[1]
HEK hH_2R	HIS	4.32 ± 0.38	4		4.96[5]	6.27[6], #
	FAM	7.75 ± 0.33	4		7.94[5]	6.87[6], #, 7.80[7], #
HEK hH_3R	HIS	6.80 ± 0.19	3		6.50[8], 6.3[9]	7.96[10], #, 7.9[9]
	PIT	8.72 ± 0.05	3		8.6[9]	8.57[11], #, 7.93[12], #, 8.0[9]
HEK hH_4R	HIS	7.25 ± 0.05	3		6.66[8], 6.8[9]	7.82[10], #, 7.9[9]]
	THIO	6.66 ± 0.12	3		6.81[8], 7.2[9]	7.42[10], #, 7.5[9]

Literature data transformed from K_i to pK_i,
The pK_i values of the cold ligands (histamine (HIS), diphenhydramine (DPH), famotidine (FAM), pitolisant (PIT) and thioperamide (THIO)) were determined on live HEK $hH_{1-4}R$ cells in the presence of 5 nM [³H]mepyramine ([³H]MEP) at hH_1R, 50 nM [³H]UR-DE257 at hH_2R, 2 nM or 5 nM [³H]UR-PI294 at hH_3R or hH_4R, respectively. Data shown are means ± sem from at least three independent experiments, each performed in triplicate.

Table S2. Radioligand competition binding experiments performed on live $\triangle G\alpha_x$ HEK $hH_{1\text{-}4}R$ cells.

		pK_i	n			pK_i	n
	hH_1R	3.37 + 0.29	4		hH_3R	6.80 + 0.19	3
$\Delta G\alpha_{s/l}$	hH_1R	< 3	4	$\Delta G\alpha_{s/l}$	hH_3R	6.92 + 0.31	3
$\Delta G\alpha_{q/11}$	hH_1R	< 3	3	$\Delta G\alpha_{q/11}$	hH_3R	7.14 + 0.22	3
$\Delta G\alpha_{12/13}$	hH_1R	2.23 + 0.37	3	$\Delta G\alpha_{12/13}$	hH_3R	6.48 + 0.04	3
$\Delta G\alpha_{six}$	hH_1R	no expression		$\Delta G\alpha_{six}$	hH_3R	6.51 + 0.08	3
	hH_2R	4.32 + 0.38	4		hH_4R	7.25 + 0.05	3
$\Delta G\alpha_{s/l}$	hH_2R	3.68 + 0.09	3	$\Delta G\alpha_{s/l}$	hH_4R	7.12 + 0.13	4
$\Delta G\alpha_{q/11}$	hH_2R	4.23 + 0.11	3	$\Delta G\alpha_{q/11}$	hH_4R	7.35 + 0.03	3
$\Delta G\alpha_{12/13}$	hH_2R	4.14 + 0.06		$\Delta G\alpha_{12/13}$	hH_4R	7.19 + 0.16	3
$\Delta G\alpha_{six}$	hH_2R	1.82 + 0.28***	3	$\Delta G\alpha_{six}$	hH_4R	7.20 + 0.16	4

The pK_i values of the cold ligands (histamine (HIS), diphenhydramine (DPH), famotidine (FAM), pitolisant (PIT) and thioperamide (THIO)) were determined on live HEK $hH_{1\text{-}4}R$ cells in the presence of 5 nM [^{3}H]mepyramine ([^{3}H]MEP) at hH_1R, 50 nM [^{3}H]UR-DE257 at hH_2R, 2 nM or 5 nM [^{3}H]UR-PI294 at hH_3R or hH_4R, respectively. Data shown are means ± sem from at least three independent experiments, each performed in triplicate. Statistical difference in pKi value among HR subtypes relative to the control (e.g. HEK hH_1R (control) versus $\triangle G\alpha_x$ HEK hH_1R) was analyzed by one-way ANOVA followed by Dunnett's multiple comparison test calculated as * $p \leq 0.05$, ** $p \leq 0.01$, *** $p \leq 0.001$, **** $p \leq 0.0001$.

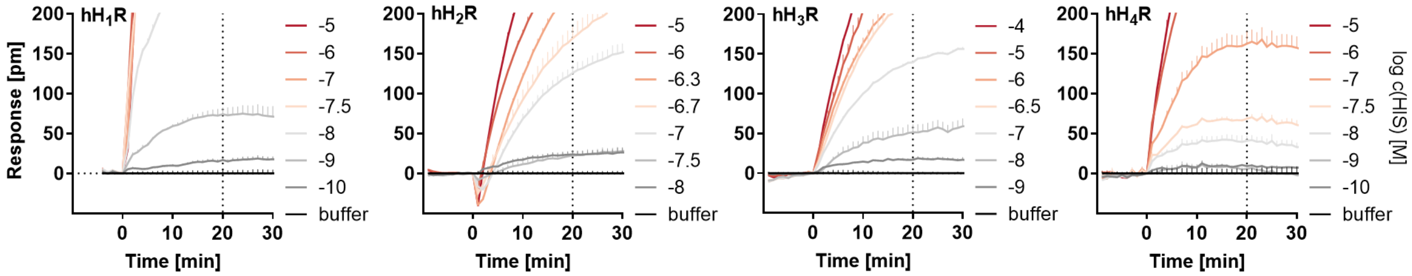

Figure S5. Zoom-in on the representative DMR traces from Figure 2A in the manuscript to highlight the signal dip observed in HEK hH_2R cells upon stimulation with HIS. Such a signal dip was not observed in HEK $hH_{1,3,4}R$ cells.

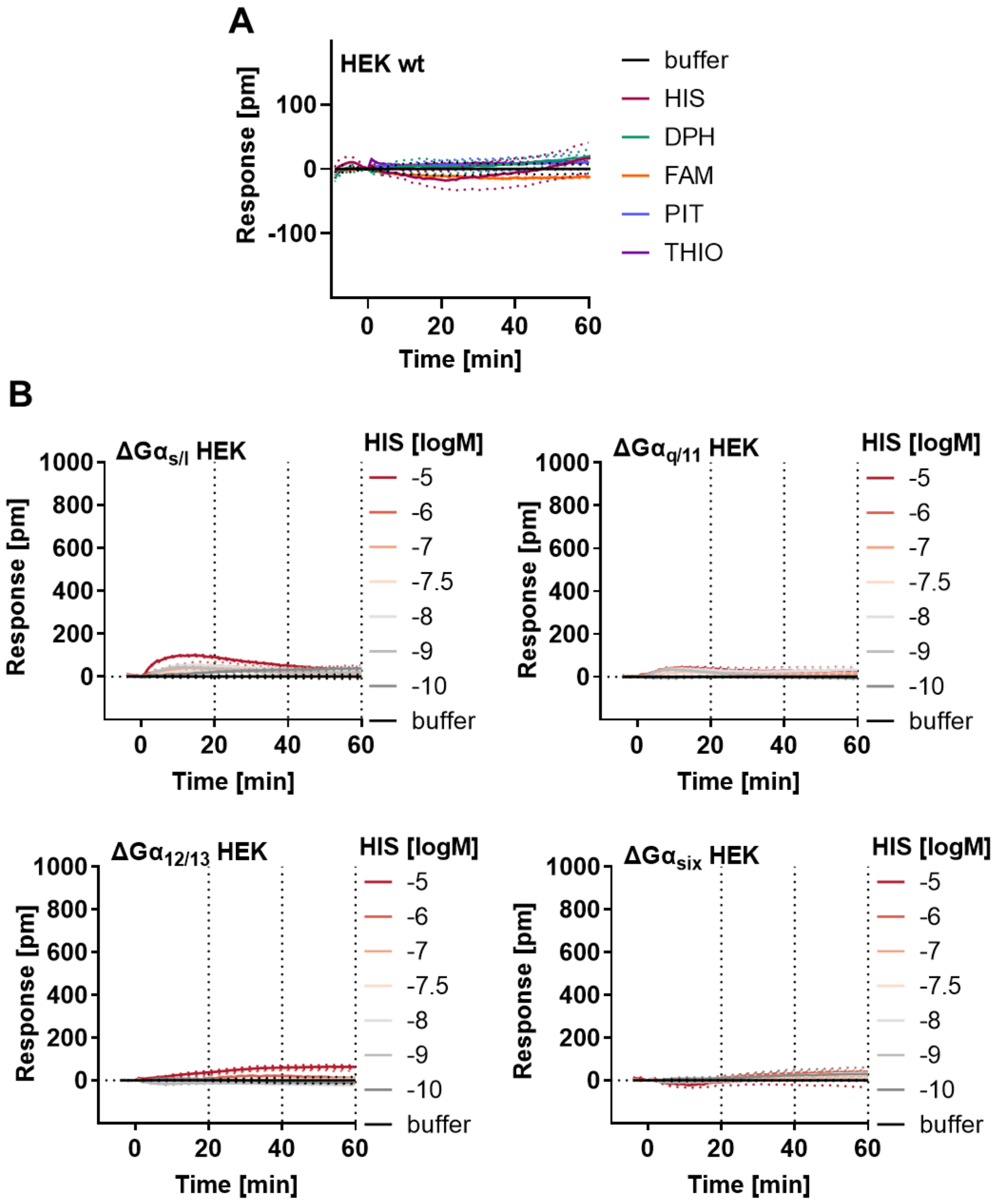

Figure S6. Lack of ligand induced DMR response in non-transfected HEK wildtype (wt) and $\Delta G\alpha_x$ HEK cells devoid hH$_{1\text{-}4}$Rs. **A**: HEK cells were stimulated with histamine (HIS), diphenhydramine (DPH), famotidine (FAM), pitolisant (PIT) and thioperamide (THIO), each at a concentration of 10 µM and the DMR response was recorded for 60 min. **B**: $\Delta G\alpha_x$ HEK cells were stimulated with increasing HIS concentration and the DMR response was recorded for 60 minutes. Shown are representative DMR traces (means ± sem) for three (**A**) or two (**B**) independent experiments, each performed in triplicate.

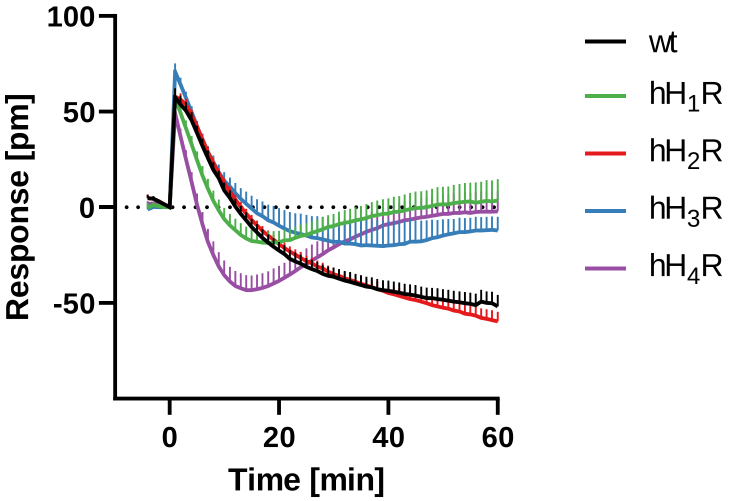

Figure S7. Constitutive activity of $hH_{1-4}Rs$ stably expressed in HEK cells. The raw DMR traces of buffer controls (in absence of a ligand) recorded using transfected HEK $hH_{1-4}R$ cells and not transfected HEK wt cells. The peaks occur immediately upon addition of the assay buffer arise due to a mechanical intervention during pipetting and are usually not visible on other DMR traces because we subtract these buffer control traces in the course of correction of the actual measured data (ligands in assay buffer). The traces presented are means ± sem of at least 10 independent experiments performed in triplicate.

Impact of the time interval used for calculations of AUC on S/B ratios

Stimulation of HEK $hH_{1\text{-}4}R$ cells with increasing histamine concentrations resulted in a positive DMR response at all 4 HR subtypes. Depending on the receptor expressed in HEK cells, the DMR responses differed with respect to the time course and amplitude (Manuscript, Figure 2A). In contrast, stimulation of $hH_{1\text{-}4}R$ cells with receptor-specific inverse agonists resulted in a negatively directed DMR response (Manuscript, Figure 2C). The quality of the signals was assessed by calculating the signal-to-background (S/B) ratio for the respective receptor-ligand combination (Manuscript, section 2.2.4). Here, the dependence of the S/B ratio on the time interval used to calculate the AUC was investigated. For this purpose, the area under curve (AUC) after 20, 40 and 60 minutes ($AUC_{20,40,60}$) for the highest histamine concentration applied in HEK $hH_{1\text{-}4}R$ cells were calculated and compared (HEK $hH_{1,2,4}R$ cells 10 µM HIS, HEK hH_3R cells 100 µM HIS). In general, high mean S/B values ranging from 123 to 323 were determined for HIS at all four receptor subtypes (Figure S8A, Table S3). In comparison to the S/B ratios determined for the agonist HIS in HEK $hH_{1\text{-}4}R$ cells, the mean S/Bs ratios determined for the inverse agonists were markedly lower ranging between 9 and 53 (Figure S8B, Table S3). With respect to HIS, the time interval used for the calculation of the AUC did not markedly affect the high S/B ratios in HEK $hH_{1,\,4}R$ cells (Figure S4A, Table S2). However, for the hH_2R and hH_3R the S/B ratios improved from AUC_{20} to AUC_{60}. An influence of the time interval used for the AUC calculation on the S/B ratio was also observed for inverse agonists (Figure S8B) showing a trend to higher S/B ratios from AUC_{20} to AUC_{60}, except for the hH_3R, where the S/B ratios remained constant. These observations are plausible when considering the time courses of the signal development (Manuscript, Figure 2A). In case of the hH_1R the maximum response was reached within 20 minutes and remained nearly on a constant level, so that the time point did not play a role for AUC calculations. This was different for HIS in HEK $hH_{2,3}R$ cells (Manuscript, Figure 2A) as well for the inverse agonists in HEK $hH_{1\text{-}4}R$ (Manuscript, Figure 2C), for which the signals developed rather slowly.

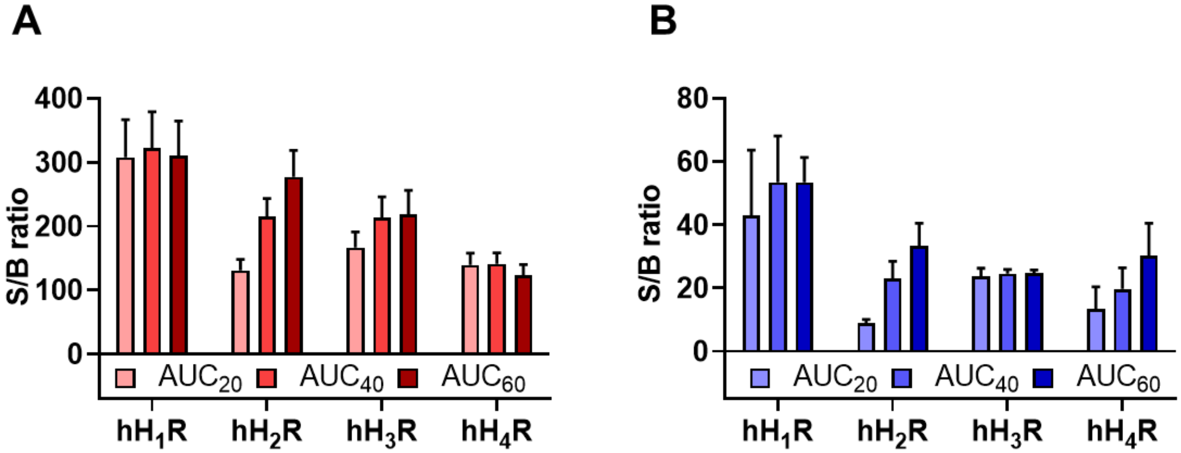

Figure S8. Impact of the time interval used for AUC calculation on the signal-to-background (S/B) ratio for histamine (red) and receptor specific inverse agonists (blue) at $hH_{1\text{-}4}Rs$. The S/B ratios were calculated from the 100% and 0% values of the respective time interval. For histamine the AUCs of the response to 10 µM histamine (100%) and buffer (0%) were used. For the inverse agonists the AUCs for 10 µM DPH at hH_1R, 10 µM FAM at hH_2R, 10 µM PIT at hH_3R and 100 µM THIO at hH_4R were used and normalized to the maximum histamine response. Data presented are means ± sem of at least three independent experiments, each performed in triplicate.

Table S3. S/B ratios determined for HIS and inverse agonists in HEK $hH_{1-4}R$ cells in the DMR assay by using AUC after 20, 40 or 60 minutes ($AUC_{20, 40, 60}$).

		AUC_{20}	AUC_{40}	AUC_{60}	n
**HEK hH_1R**	HIS	308 ± 59	323 ± 56	310 ± 54	14
	DPH	43 ± 21	53 ± 15	53 ± 8	3
**HEK hH_2R**	HIS	131 ± 17	215 ± 28	277 ± 41	23
	FAM	9 ± 1	23 ± 5	33 ± 7	3
**HEK hH_3R**	HIS	167 ± 23	213 ± 33	218 ± 38	7
	PIT	24 ± 3	25 ± 1	25 ± 1	3
**HEK hH_4R**	HIS	139 ± 18	141 ± 17	123 ± 17	25
	THIO	13 ± 7	20 ± 7	30 ± 10	3

The S/B ratios were calculated from the 100% and 0% values of the respective time interval. For histamine the AUCs of the response to 10 µM histamine (100%) and buffer (0%) were used. For the inverse agonists the AUCs for 10 µM DPH (diphenhydramine) at hH_1R, 10 µM FAM (famotidine) at hH_2R, 10 µM PIT (pitolisant) at hH_3R and 100 µM THIO (thioperamide) at hH_4R were used and normalized to the maximum histamine response. Data presented are means ± sem of n independent experiments, each performed in triplicate.

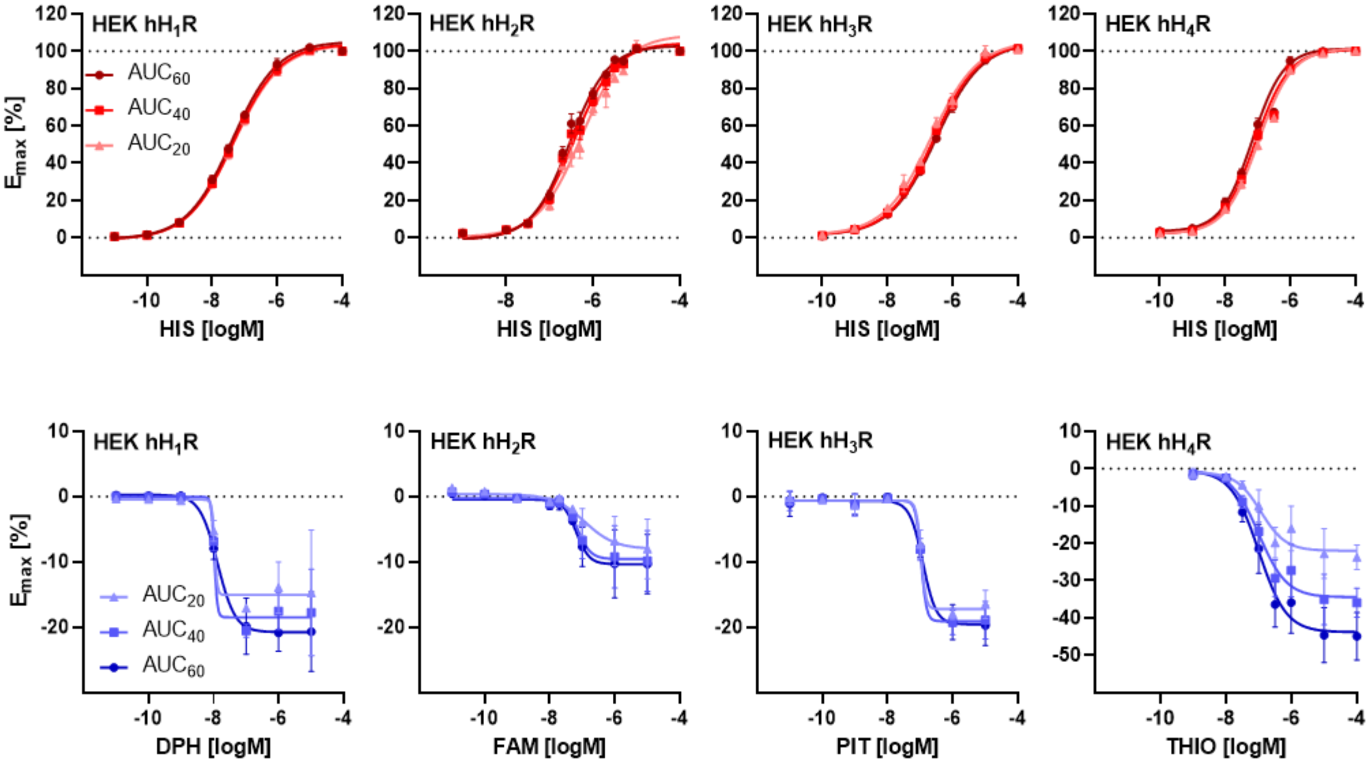

Figure S9. CRCs constructed for histamine (HIS, red) and inverse agonists (blue) in HEK hH$_{1\text{-}4}$R cells in the DMR assay by using AUC after 20, 40 or 60 minutes (AUC$_{20, 40, 60}$). HEK hH$_{1\text{-}4}$R cells were stimulated either with HIS or a specific inverse agonist and the DMR response was recorded for 60 minutes. Inverse agonists were abbreviated as follows: DPH (diphenhydramine), FAM (famotidine), PIT (pitolisant) and THIO (thioperamide). For conversion of the DMR responses to CRCs the AUC$_{20, 40, 60}$ was used. Each point represents mean ± sem of at least three independent experiments, each performed in triplicate.

Table S4. The pEC_{50} and E_{max} values determined for HIS and inverse agonists determined in the DMR assay in HEK $hH_{1-4}R$ cells by using AUC after 20, 40 or 60 minutes ($AUC_{20, 40, 60}$).

		AUC_{20}		AUC_{40}		AUC_{60}	
		**pEC_{50}**	**E_{max} [%]**	**pEC_{50}**	**E_{max} [%]**	**pEC_{50}**	**E_{max} [%]**
HEK hH_1R	HIS	7.32 ± 0.08	100	7.33 ± 0.06	100	7.43 ± 0.05	100
	DPH	7.74 0.04	-15.4 ± 4.8	7.86 ± 0.04	-18.6 ± 3.3	7.82 ± 0.07	-21.9 ± 3.0
HEK hH_2R	HIS	6.30 ± 0.05	100	6.47 ± 0.05	100	6.57 ± 0.05	100
	FAM	6.99 ± 0.18	-8.2 ± 4.7	7.30 ± 0.04	-9.6 ± 4.9	7.37 ± 0.06	-10.3 ± 5.0
HEK hH_3R	HIS	6.66 ± 0.07	100	6.57 ± 0.05	100	6.49 ± 0.06	100
	PIT	6.88 ± 0.07	-18.0 ± 1.4	6.86 ± 0.08	-19.6 ± 2.3	7.02 ± 0.22	-20.5 ± 2.5
HEK hH_4R	HIS	6.99 ± 0.05	100	7.08 ± 0.05	100	7.15 ± 0.05	100
	THIO	7.04 ± 0.18	-20.1 ± 5.0	7.06 ± 0.13	-32.0 ± 5.3	7.04 ± 0.14	-45.0 ± 5.7

HEK $hH_{1-4}R$ cells were stimulated either with HIS or a specific inverse agonist and the DMR response was recorded for 60 minutes. Inverse agonists were abbreviated as follows: DPH (diphenhydramine), FAM (famotidine), PIT (pitolisant) and THIO (thioperamide). For conversion of the DMR responses to CRCs $AUC_{20, 40, 60}$ was used. Data presented are means ± sem of at least three independent experiments, each performed in triplicate.

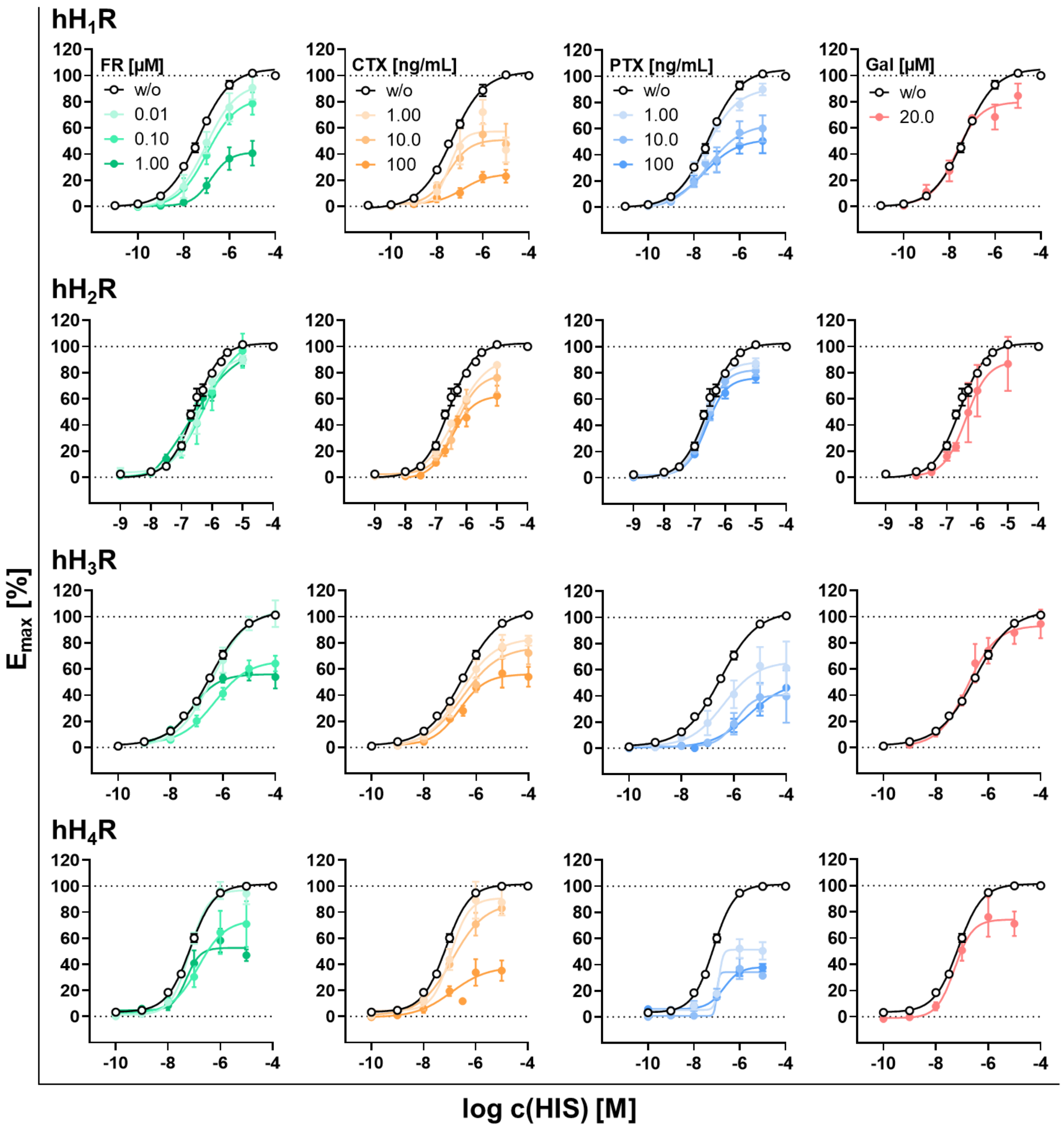

Figure S10. CRCs determined for histamine (HIS) using HEK hH1-4R cells in the absence and the presence of G protein modulators. HEK hH1-4R cells were pre-treated with indicated concentrations of the respective G protein inhibitor FR (green), PTX, (blue), CTX (orange) and gallein (red). The CRCs were constructed by calculating the AUC60 for the DMR traces recorded in the absence and the presence of G protein modulators at increasing HIS concentrations. Each point represents mean ± sem of at least three independent experiments, each performed in triplicate.

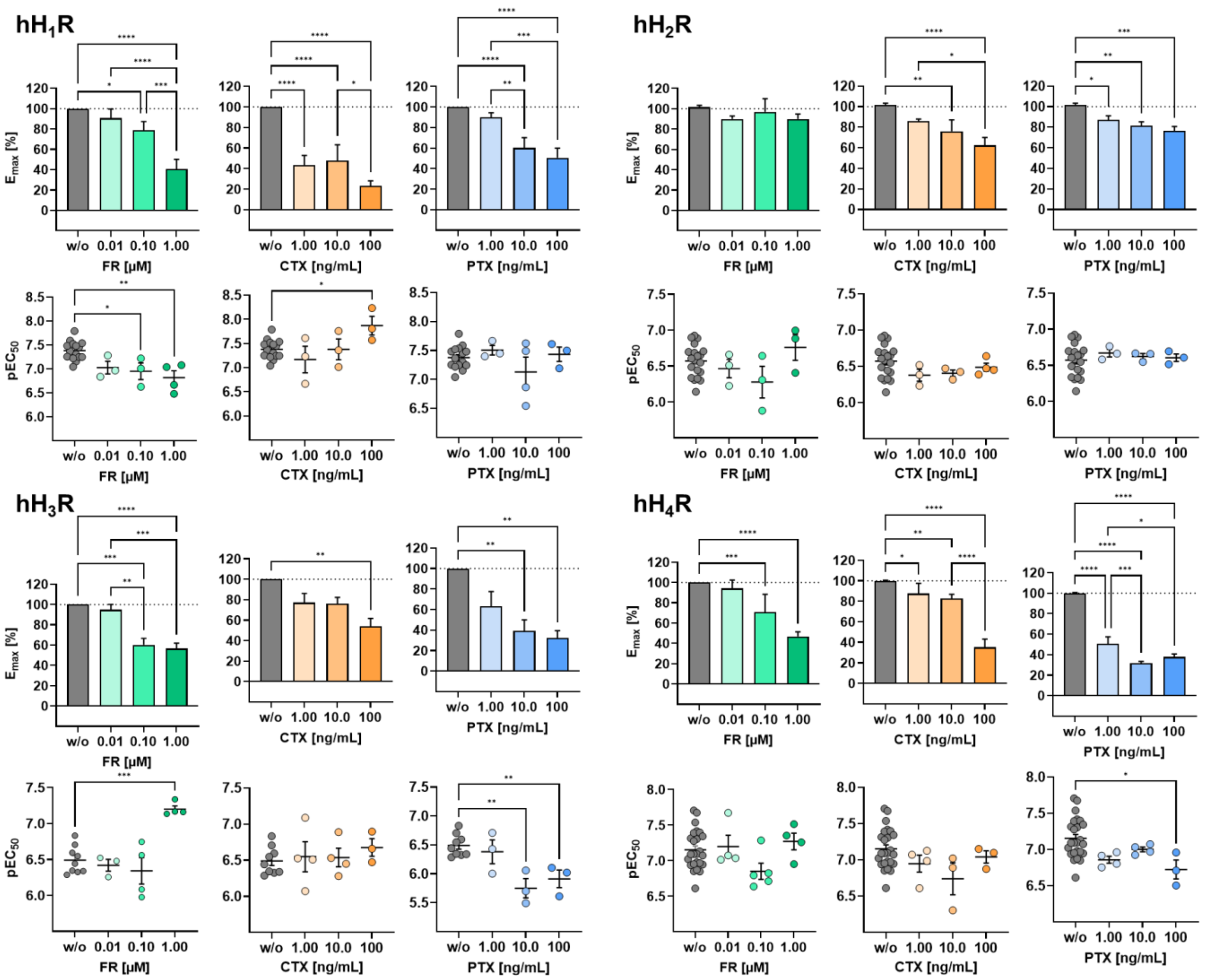

Figure S11. E_{max} and pEC_{50} values determined for HIS in the absence and the presence of G protein modulators. The E_{max} values determined for HIS in the absence (grey) and the presence of FR (green), CTX (orange) and PTX (blue). Bar charts show the E_{max} values were calculated using AUC_{60} for at the highest HIS concentration (10 µM for $hH_{1,2,4}R$ and 100 µM for hH_3R) and normalized to the AUC_{60} of the untreated control (100%) and buffer value (0%). The scatter plots show the pEC_{50} values determined in absence (grey) and presence of G protein modulators at the concentration stated above. The pEC_{50} were determined by plotting the AUC_{60} against the respective HIS concentration. Data presented are means ± sem of at least three independent experiments, each performed in triplicate. Statistical differences were analyzed by one-way ANOVA followed by Tukeys's multiple comparison and are indicated as asterisks (* $p \leq 0.05$, ** $p \leq 0.01$, *** $p \leq 0.001$, **** $p \leq 0.0001$).

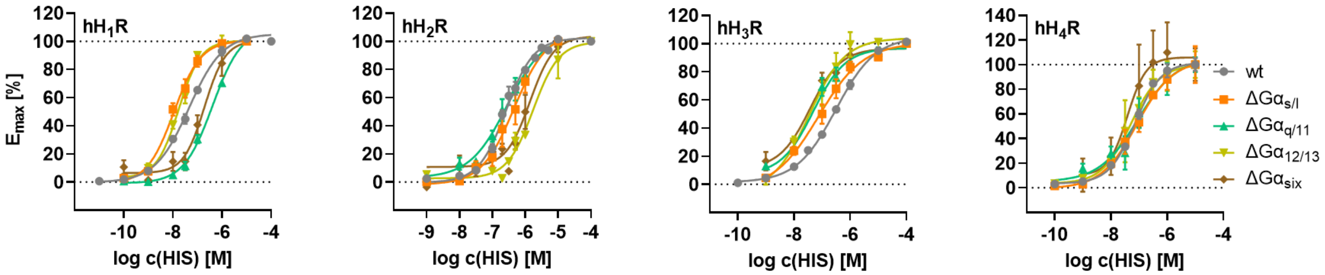

Figure S12. CRCs determined for histamine (HIS) using $\Delta G\alpha_x$ HEK $hH_{1\text{-}4}R$ cells The CRCs were constructed by calculating the AUC_{60} for the DMR traces recorded at increasing HIS concentrations. The AUC_{60} at the respective HIS concentration ($hH_{1,2,4}Rs$ 10 µM HIS and hH_3R 100 µM HIS) using $\Delta G\alpha_x$ HEK $hH_{1\text{-}4}R$ cells was normalized to the mean AUC_{60} of HEK $hH_{1\text{-}4}R$ cells, in which all G proteins were present (100% control). This approximation was reasonable, because mostly the expression of the different receptor subtypes was comparable (Manuscript, Table 1). Each point represents mean ± sem of at least three independent experiments, each performed in triplicate.

References

1. Bakker, R. A.; Schoonus, S. B.; Smit, M. J.; Timmerman, H.; Leurs, R., Histamine H(1)-receptor activation of nuclear factor-kappa B: roles for G beta gamma- and G alpha(q/11)-subunits in constitutive and agonist-mediated signaling. *Mol Pharmacol* **2001,** 60, (5), 1133-42.
2. Pertz, H. H.; Gornemann, T.; Schurad, B.; Seifert, R.; Strasser, A., Striking differences of action of lisuride stereoisomers at histamine H_1 receptors. *Naunyn-Schmiedeberg's archives of pharmacology* **2006,** 374, (3), 215-22.
3. Moguilevsky, N.; Varsalona, F.; Noyer, M.; Gillard, M.; Guillaume, J. P.; Garcia, L.; Szpirer, C.; Szpirer, J.; Bollen, A., Stable expression of human H1-histamine-receptor cDNA in Chinese hamster ovary cells. Pharmacological characterisation of the protein, tissue distribution of messenger RNA and chromosomal localisation of the gene. *Eur J Biochem* **1994,** 224, (2), 489-95.
4. Lieb, S.; Littmann, T.; Plank, N.; Felixberger, J.; Tanaka, M.; Schafer, T.; Krief, S.; Elz, S.; Friedland, K.; Bernhardt, G.; Wegener, J.; Ozawa, T.; Buschauer, A., Label-free versus conventional cellular assays: Functional investigations on the human histamine H1 receptor. *Pharmacological research* **2016,** 114, 13-26.
5. Gratz, L.; Tropmann, K.; Bresinsky, M.; Muller, C.; Bernhardt, G.; Pockes, S., NanoBRET binding assay for histamine H2 receptor ligands using live recombinant HEK293T cells. *Sci Rep* **2020,** 10, (1), 13288.
6. Baumeister, P.; Erdmann, D.; Biselli, S.; Kagermeier, N.; Elz, S.; Bernhardt, G.; Buschauer, A., [(3) H]UR-DE257: development of a tritium-labeled squaramide-type selective histamine H2 receptor antagonist. *ChemMedChem* **2015,** 10, (1), 83-93.
7. Leurs, R.; Smit, M. J.; Menge, W. M.; Timmerman, H., Pharmacological characterization of the human histamine H2 receptor stably expressed in Chinese hamster ovary cells. *Br J Pharmacol* **1994,** 112, (3), 847-54.
8. Bartole, E.; Grätz, L.; Littmann, T.; Wifling, D.; Seibel, U.; Buschauer, A.; Bernhardt, G., UR-DEBa242: A Py-5-Labeled Fluorescent Multipurpose Probe for Investigations on the Histamine H3 and H4 Receptors. *Journal of medicinal chemistry* **2020**.
9. Mocking, T. A. M.; Verweij, E. W. E.; Vischer, H. F.; Leurs, R., Homogeneous, Real-Time NanoBRET Binding Assays for the Histamine H3 and H4 Receptors on Living Cells. *Mol Pharmacol* **2018,** 94, (6), 1371-1381.
10. Igel, P.; Schnell, D.; Bernhardt, G.; Seifert, R.; Buschauer, A., Tritium-labeled N(1)-[3-(1H-imidazol-4-yl)propyl]-N(2)-propionylguanidine ([(3)H]UR-PI294), a high-affinity histamine H(3) and H(4) receptor radioligand. *ChemMedChem* **2009,** 4, (2), 225-31.
11. Ligneau, X.; Perrin, D.; Landais, L.; Camelin, J. C.; Calmels, T. P.; Berrebi-Bertrand, I.; Lecomte, J. M.; Parmentier, R.; Anaclet, C.; Lin, J. S.; Bertaina-Anglade, V.; la Rochelle, C. D.; d'Aniello, F.; Rouleau, A.; Gbahou, F.; Arrang, J. M.; Ganellin, C. R.; Stark, H.; Schunack, W.; Schwartz, J. C., BF2.649 [1-{3-[3-(4-Chlorophenyl)propoxy]propyl}piperidine, hydrochloride], a nonimidazole inverse agonist/antagonist at the human histamine H3 receptor: Preclinical pharmacology. *The Journal of pharmacology and experimental therapeutics* **2007,** 320, (1), 365-75.
12. Sander, K.; Kottke, T.; Weizel, L.; Stark, H., Kojic acid derivatives as histamine H(3) receptor ligands. *Chem Pharm Bull (Tokyo)* **2010,** 58, (10), 1353-61.
